# Hepatoprotection of Probiotics Against Non-Alcoholic Fatty Liver Disease *in vivo*: A Systematic Review

**DOI:** 10.3389/fnut.2022.844374

**Published:** 2022-04-11

**Authors:** Faezah Sabirin, Siong Meng Lim, Chin Fen Neoh, Kalavathy Ramasamy

**Affiliations:** ^1^Collaborative Drug Discovery Research (CDDR) Group, Faculty of Pharmacy, Universiti Teknologi MARA (UiTM), Cawangan Selangor, Kampus Puncak Alam, Bandar Puncak Alam, Malaysia; ^2^Centre of Preclinical Science Studies, Faculty of Dentistry, Universiti Teknologi MARA (UiTM), Cawangan Selangor, Kampus Sungai Buloh, Sungai Buloh, Malaysia

**Keywords:** probiotics, non-alcoholic fatty liver disease, rodents, histopathology, dysbiosis

## Abstract

Probiotic supplements have been increasingly reported for their usefulness in delaying the development and progression of non-alcoholic fatty liver disease (NAFLD). Literature on the impact of probiotics on NAFLD covered various aspects of the disease. This study was undertaken to systematically review *in vivo* findings on hepatoprotection of probiotics against NAFLD. The literature search was performed through Cochrane, PubMed/MEDLINE, Embase, and Web of Science databases. Interventions of known probiotics in NAFLD-induced animal model with at least one measurable NAFLD-related parameter were included. The data were extracted by all authors independently. Quality assessment was conducted using the Systematic Review Center for Laboratory animal Experimentation (SYRCLE's) Risk of Bias (RoB) tool. *P*-values of measures were compared inter- and intra-study for each parameter. Forty-four probiotic-based studies of NAFLD-induced rodents were shortlisted. The majority of the studies were presented with low/unclear risk of bias. Probiotics improved the histopathology of NAFLD rodents (primary outcome). Most of the probiotic-supplemented NAFLD rodents were presented with mixed effects on serum liver enzymes but with improved hepatic and serum lipid profiles (including increased serum high-density lipoprotein cholesterol). The findings were generally accompanied by downregulation of hepatic lipogenic, oxidative, and inflammatory signallings. Probiotics were found to modulate gut microbiota composition and its products, and intestinal permeability. Probiotics also resulted in better glycaemic control and reduced liver weight. Altogether, the present qualitative appraisals strongly implied the hepatoprotective potential of probiotics against NAFLD *in vivo*.

## Introduction

Non-alcoholic fatty liver disease (NAFLD) is a major public health issue that affects all ages and accounts for a pooled prevalence of 25% worldwide (1). It is characterized by liver histological phenotypes and other accompanying metabolic comorbidities such as obesity, dyslipidaemia, and diabetes mellitus (2). The burden of NAFLD is associated with increased risk of extrahepatic diseases including cardiovascular diseases, chronic kidney disease, and cancers (i.e., colorectal and breast cancers) (3, 4).

Being a multifactorial disease, NAFLD is often described as “multiple parallel hits” that involve the host's genetics, immune system, epigenetics (5), and derangement of the gut-liver-adipose axis (6, 7). Excessive nutrient intake, which is associated with dysbiosis (5, 8, 9), acts as one of the major risk factors of NAFLD (10, 11) that could lead to elevated free fatty acid (FFA) and cholesterol, mitochondrial damage (12), insulin resistance (13), adipocyte dysfunctions and oxidative stress (6, 14). Dysbiosis is also linked to disruption of the intestinal barrier integrity, which further increases the delivery of gut-derived insults to the liver (5, 15), initiating inflammatory responses (16).

To date, there is no FDA-approved drug therapy against NAFLD. Whilst biopsy-confirmed NASH patients are recommended with vitamin E (800 I.U.) or pioglitazone (17), the latter, a thiazolidinedione derivative, offers some degree of liver histopathology improvements (18). There is also increasing evidence on the use of probiotics as alternative hepatoprotective agents against NAFLD. The rationale of this approach lies in their ability in restoring gut homeostasis (5), which in turn could improve lipid metabolism through the gut-liver axis.

The use of *in vivo* models in preclinical studies offers a thorough examination of the liver not only through histopathological evaluation but also through the determination of hepatic lipid profiles, gene and protein expressions, and systemic and inter-organ effects. Unfortunately, these parameters are not routinely assessed in all NAFLD patients as these assessments require liver sampling through biopsy (17). The present systematic review appraised findings of all *in vivo* studies that fulfilled the inclusion criteria. In general, qualitative appraisal such as this is reliable in preventing biased reporting of research findings, recognizing proper methodological approach (19, 20), and uncovering possible relationships and mechanisms underlying prevention of NAFLD by probiotics *in vivo*. The pooled information from the various *in vivo* studies that covered a diverse range of disease models, research groups, and study characteristics would allow the drawing of conclusions that are relatively more reflective of the issues of human disease condition (19), whilst minimizing unnecessary duplication of animal studies (21).

## Methods

### Literature Search Strategy

This systematic review was conducted in accordance with the Preferred Reporting Items for Systematic Reviews and Meta-analyses (PRISMA) guidelines (22). The search for published articles was performed through four electronic databases, namely, Cochrane, EMBASE, PubMed/Medline, and Web of Science. The Boolean search involved the combined use of the terms such as “non-alcoholic fatty liver disease”, “NAFLD”, “fatty liver”, “non-alcoholic steatohepatitis”, “NASH”, “probiotic”, “prebiotic”, “symbiotic”, “lactobacilli”, “bifidobacter”, and “flora”.

### Study Selection

The inclusion criteria of this systematic review included (a) diet-induced or genetically induced NAFLD or NASH animal models; (b) probiotic intervention(s); (c) fermented food with known probiotic genus(genera)/ strain(s); (d) at least one of the following measurable parameters was assessed: liver histology (steatosis with or without inflammation), liver function tests [including liver enzymes, i.e., serum alanine aminotransferase (ALT), aspartate aminotransferase (AST), alkaline phosphatase (ALP), and gamma-glutamyl transferase (GGT), and serum bilirubin], lipid parameters [hepatic and serum profiles including lipid, triacylglycerol (TAG), total cholesterol (TC), low-density lipoprotein cholesterol (LDL-C), high-density lipoprotein cholesterol (HDL-C), esterified cholesterol, free cholesterol, phospholipid (PL), and non-esterified fatty acid (NEFA)], oxidative markers [e.g., malondialdehyde (MDA) and transforming growth factor beta (TGF-β)], inflammatory markers [e.g., tumor necrosis factor alpha (TNF-α), interleukin-6 (IL-6), interleukin 1beta (IL-1β), and necrosis factor kappa B (NF-κB)], glycaemic parameters [i.e., fasting blood glucose (FBG), fasting insulin, and homeostasis model assessment of insulin resistance (HOMA-IR)], body or organ weight and gut related changes [i.e., gut microbiota, gut-derived products [i.e., endotoxins, lipopolysaccharide (LPS), short-chain fatty acid (SCFA)], and intestinal permeability]. The exclusion criteria included (a) animal models of < 97% genomic similarities to human (e.g., rooster and fish); (b) *in vivo* studies of other diseases that caused steatosis or steatofibrosis (e.g., viral hepatitis and carcinogenesis); (c) combination of probiotics with other interventions (e.g., bioactive compounds, drugs, and herbs); (d) pre- or synbiotics; (e) abstracts or conference proceedings, *in vitro* studies, clinical studies, review papers, and non-English literature; and (f) use of secondary data from other published works. The eligibility of all potential studies identified for inclusion was independently assessed by four authors (i.e., FS, KR, SML, and CFN). Discrepancies on study inclusion were resolved through discussion and consensus.

### Data Extraction

Data from the shortlisted studies (i.e., types of intervention, inducement of NAFLD, age of the animal model, details of the probiotics used, duration of intervention, dosage forms, baseline, and post-intervention values of each parameter) were obtained independently and extracted by the authors (FS, KR, SML, and CFN) using a standardized, electronic extraction form.

### Assessment of the Risk of Bias Using the SYRCLE's RoB Tool

The risks of bias of the shortlisted studies were assessed by all authors using the Systematic Review Center for Laboratory animal Experimentation (SYRCLE's) Risk of Bias (RoB) tool that was adapted specifically for animal-based intervention studies (20). The assessment categories included selection, performance, detection, attrition, reporting, and other possible sources of biases (see Appendix A in Supplementary Material). The studies were deemed as low risk of bias when the total number of high risks by categories <3, moderate risk of bias when the total number of high risks by categories >3 but <6, or high risk of bias when the total number of high risks by categories >6. Disagreement was resolved by discussion.

## Results

### Literature Search Outcomes and Characteristics of Shortlisted *in vivo* Studies

**Figure 1:**
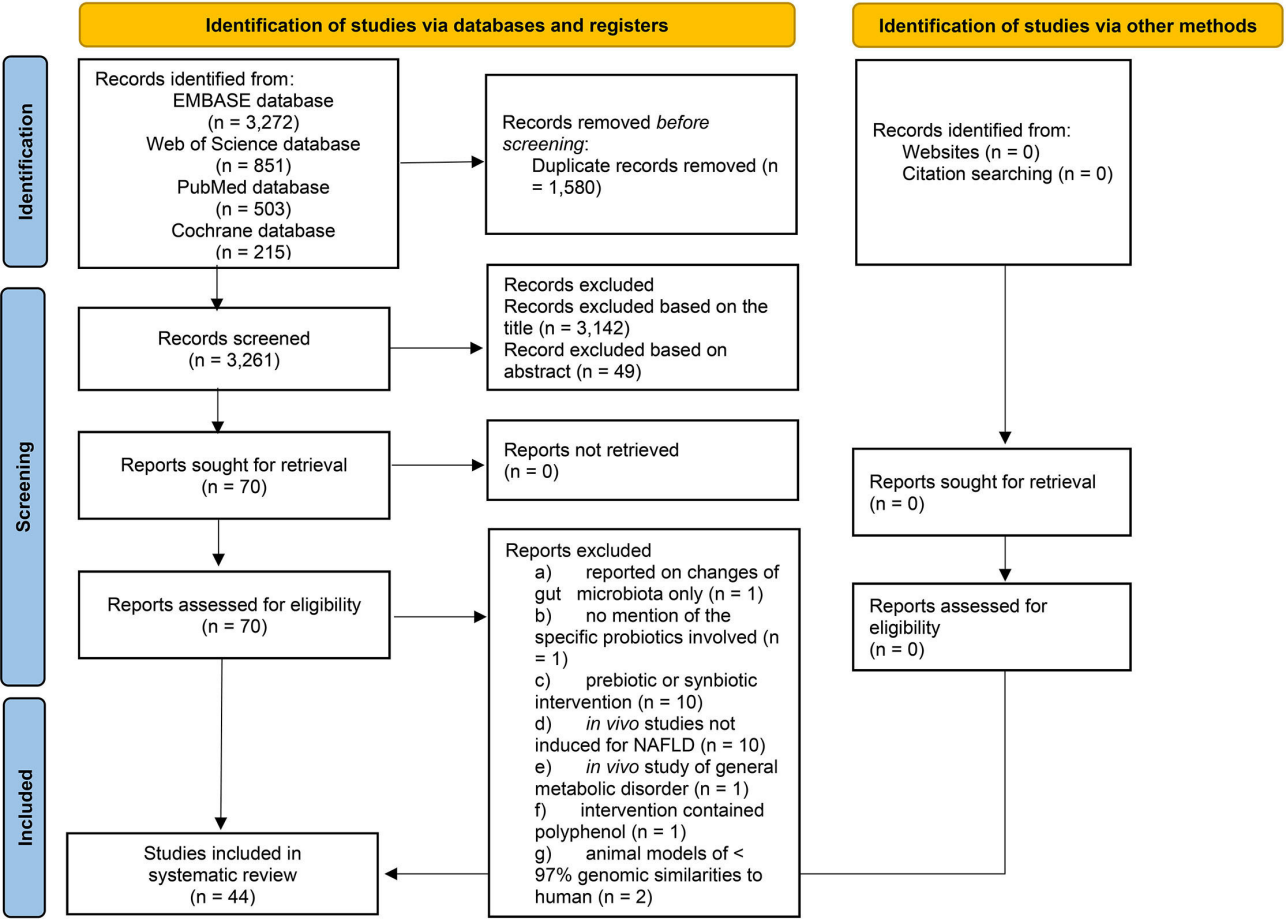
PRISMA flowchart of the literature selection process of the present systematic review.

This literature search had yielded publications between 1966 and March 2020. The PRISMA flowchart (Figure 1) shows that out of the 4,841 studies identified through electronic search, 44 studies were included in the qualitative synthesis. Appendix B highlights the findings from all 44 shortlisted studies (see Appendix B in Supplementary Material); Please note that some included studies reported findings of more than one interventions.

### Risk of Bias of Shortlisted *in vivo* Studies

**Figure 2:**
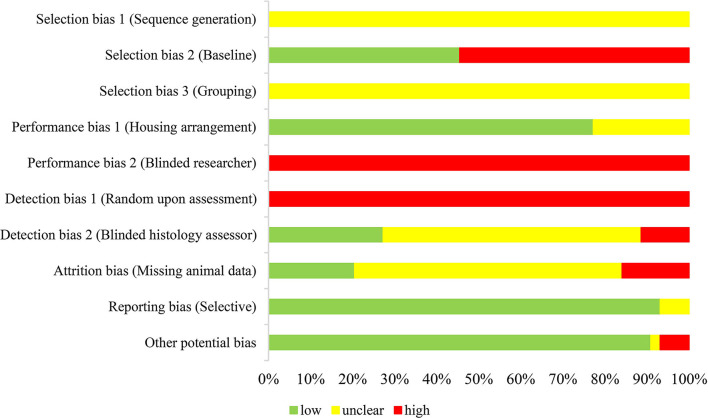
Summary of outcomes derived from the SYRCLE's RoB risk of bias assessment tool (*n* = 44).

This systematic review had utilized the SYRCLE's RoB tool adapted from the Cochrane risk of bias tool suited for animal intervention study (20) to evaluate the quality of the shortlisted studies. Figure 2 shows that the shortlisted studies were presented with either low or unclear risks of bias under most of the assessment categories. All studies, however, elicited high risks of bias in the aspects of “*evidence of researcher blinded to the intervention*” (performance bias 2; blinded researcher) and “*selection during assessment were at random*” (detection bias 1; assessment). Besides, all shortlisted studies were presented with unclear risks of bias for sequence generation and grouping. There was no mention as to whether sequence allocation was used to produce comparable groups and no description on assignment of groups. Additionally, 24 studies (55%) were presented with high risk on selection bias 2 (baseline characteristics), whereby the baseline details of the rodents like gender, age, and initial body weight were not disclosed. On the contrary, the majority of the shortlisted studies (77%) were presented with a low risk of bias for housing arrangement, in which the present assessment defined housing arrangement as identical housing conditions in which all animals were kept in an area with controlled temperature and lighting. The remaining studies were presented with unclear risk of bias as no details on housing arrangement were mentioned. Besides, seven studies (16%) showed a high risk on attrition bias given the unexplained missing of *n* number which was detected from inconsistent number of animals mentioned in the “Methods” and “Results” sections. Five studies (11%) were deemed having high risk of detection bias 2 (blinded histology assessor) as liver histology was not assessed. The majority of the included studies were, however, presented with unclear risk of bias as they did not specify as to whether the histology assessments were conducted in a blinded manner. On another note, there were three studies (7%) that elicited high risks of other potential biases related to reporting or editorial errors. One of them was due to an inappropriate experimental design whereby probiotic-supplemented HFD-induced NAFLD mice were compared with normal chow-fed mice instead of HFD-induced NAFLD mice. Nevertheless, this particular study by Jang, Park (23) was still included in this systematic review (see Appendix B in Supplementary Material) as it fulfilled the inclusion criteria. In general, this assessment did not detect selective reporting in most of the shortlisted studies as the report of findings corresponded well to the methods that were appropriately aligned to the respective objectives.

### Primary Outcome: Probiotics Reduced the Severity of Liver Histopathology in NAFLD *in vivo*

**Figure 3:**
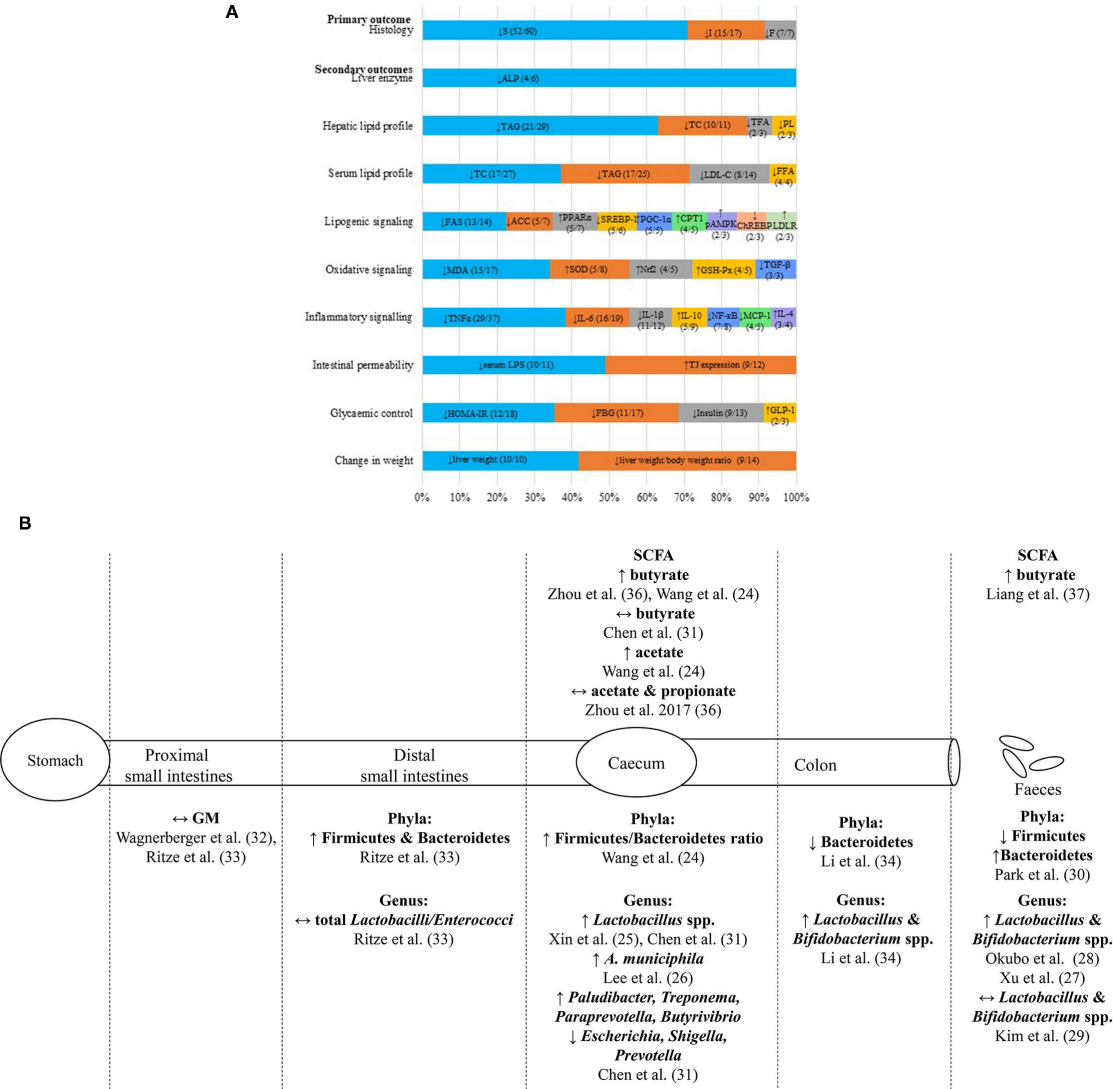
Summary of hepatoprotective effects of probiotic supplementations on NAFLD *in vivo*. **(A)** The infographic represents the commonly reported biochemical and molecular parameters (>2 studies) with the most reported trend of significant findings (>50% studies; *p* < 0.05) of NAFLD animal models supplemented with probiotics. Each horizontal stacked column represents the total number of findings which had reported the common parameters of the included studies. The number in each data series of each stacked column represents the most reported trend of significant findings (>50%; *p* < 0.05) out of the total number of findings reported in the included studies on each parameter. **(B)** The illustration represents the reported changes of gut microbiota and SCFA compositions at various sites along the gut of NAFLD animal models supplemented with probiotics. ACC, Acetyl-CoA carboxylase; ALP, alkaline phosphatase; *A. municiphila, Akkermansia miniciphila*; ChREBP, carbohydrate response-element binding protein; CPT1, carnitine palmitoyltransferase I; *E. coli, Escherichia coli*; F, fibrosis; FAS, fatty acid synthase; FBG, fasting blood glucose; GLP-1, glucagon-like peptide-1; GM, gut microbiota; GSH-Px, glutathione peroxidase; HOMA-IR, homeostasis model assessment for insulin resistance; I, inflammation; IL-1β, interleukin-1; IL-4, interleukin-4; IL-6, interleukin-6; IL-10, interleukin-10; HDL-C, high-density lipoprotein cholesterol; LDL-C, low-density lipoprotein cholesterol; LDLR, low-density lipoprotein receptor; LPS, lipopolysaccharide; MDA, malanoyl dialdehyde; NF-κB, necrosis factor kappa B; Nrf2, nuclear factor erythroid 2-related factor 2; pAMPK, phosphorylated AMP-activated protein kinase; PL, phospholipid; PPARɤ, peroxisome proliferator-activated receptor alpha; PGC-1ɑ, peroxisome proliferator-activated receptor gamma coactivator 1 alpha; S, steatosis; SCFA, short chain fatty acid; SOD, superoxide dismutase; SREBP-1, sterol regulatory element-binding protein 1; TAG, triacylglycerol; TC, total cholesterol; TFA, total fatty acid; TGF-α, transforming growth factor beta; TJ, tight junction protein; TLR4, toll-like receptor 4; TNF-α, tumor necrosis factor alpha; ↑, increased; ↓, reduced; ↔, no changes.

This qualitative appraisal of the shortlisted studies on liver histopathology supported the beneficial use of probiotic supplementations in halting disease progression of NAFLD *in vivo*. Probiotic supplementations reportedly reduced the severity of liver histopathology in NAFLD rodents, with characteristics of decreased steatosis (*n* = 52/59), inflammation (*n* = 15/17), and fibrosis (*n* = 7/7) (Figure 3A). There were 24 probiotic-based studies that had used the total NAFLD activity score (NAS) as the scoring system for the severity of liver histopathology (see Appendix B in Supplementary Material for details of probiotics and findings of each study).

### Secondary Outcomes: Biochemical and Molecular Parameters

#### Probiotics Yielded Mixed Effects on Liver Function in NAFLD *in vivo*

The qualitative appraisal revealed that the probiotic-supplemented NAFLD rodents were presented with reduced serum ALP (*n* = 4/6) (Figure 3A), with mixed outcomes on serum ALT (reduced; *n* = 25/52, no changes; *n* = 26/52, increased; *n* = 1/52), and with no changes on serum AST (*n* = 27/38). As for serum bilirubin (i.e., total, direct, and indirect), the majority of the included studies reported no significant changes of this parameter despite the supplementation of probiotics (see Appendix B in Supplementary Material for details of probiotics and findings of each study).

#### Probiotics Generally Reduced Hepatic and Common Serum Lipid Profiles and Downregulated Hepatic Lipogenic Signaling in NAFLD *in vivo*

The qualitative appraisal revealed trends of reduced hepatic [TAG (*n* = 21/29), TC (*n* = 10/11), total fatty acid (*n* = 2/3), and phospholipid (PL) (*n* = 2/3)] and serum [TAG (*n* = 17/25), TC (*n* = 17/27), LDL-C (*n* = 8/14), and FFA (*n* = 4/4)] lipid profiles in probiotic-supplemented NAFLD rodents (Figure 3A). For the regulation of the lipid metabolism, most of the included probiotic-based *in vivo* studies reported downregulation of the lipogenic signaling through reduced hepatic fatty acid synthase (FAS) (*n* = 13/14), acetyl-CoA carboxylase (ACC) (*n* =5/7), sterol regulatory element-binding protein 1 (SREBP-1) (*n* = 5/6), and carbohydrate response-element binding protein (ChREBP) (*n* = 2/3) and/or increased fatty acid receptor peroxisome proliferator-activated receptor alpha (PPARα) (*n* = 5/7), PPAR gamma (PPARα) coactivator 1 alpha (PGC-1α) (*n* = 5/5), carnitine palmitoyltransferase I (CPT1) (*n* = 4/5), AMP-activated protein kinase (AMPK) phosphorylation (*n* = 2/3) and low-density lipoprotein receptor (LDLR) (*n* = 2/3) (Figure 3A) (see Appendix B in Supplementary Material for details of probiotics and findings of each study).

#### Probiotics Generally Downregulated Oxidative and Inflammatory Responses in NAFLD *in vivo*

Oxidative signaling markers were downregulated in probiotic-supplemented NAFLD rodents. Previous studies reported reduced MDA (*n* = 15/17) and TGF-β (*n* = 3/3) and/or increased SOD (*n* = 5/8), nuclear factor Nrf2 (*n* = 4/5) and glutathione peroxidase (GSH-Px) (*n* = 4/5) (Figure 3A). The inflammatory signaling pathways were also downregulated in probiotic-supplemented NAFLD rodents, mainly through the downregulation of serum or hepatic TNF-α (*n* = 29/37), IL-6 (*n* = 16/19), IL-1β (*n* = 11/12), NF-κB (*n* = 7/8), and monocyte chemotactic and activating factor 1 (MCP-1) (*n* = 4/5), and upregulation of interleukin 10 (IL-10) (*n* = 5/9) and interleukin 4 (IL-4) (*n* = 3/4) (Figure 3A) (see Appendix B in Supplementary Material for details of probiotics and finding of each study).

#### Probiotics Generally Improved Dysbiosis and Gut Permeability and Altered SCFA Level in NAFLD *in vivo*

The shortlisted probiotic studies also assessed the differential gut microbiota composition and SCFA from various sites along the gut of NAFLD animal models (Figure 3B). Most of the findings were from samples of the caecum (24–26) and excreted feces (27–30). The caecal contents of probiotic-supplemented NAFLD rodents were presented with increased *Lactobacillus* spp. (25, 31) (Figure 3B). Whilst the fecal samples of probiotic-fed NAFLD rodents were presented with increased *Lactobacillus* and *Bifidobacterium* spp. (27, 28), samples from the proximal and distal small intestines exhibited no differential changes in the gut microbiota composition (32, 33) (Figure 3B). As for colonic content, Li, Nie (34) reported on increased *Lactobacillus* and *Bifidobacterium* spp. (Figure 3B). Additionally, three studies (35–37) reported increased butyrate in caecal or fecal contents of probiotic-supplemented NAFLD rodents. On another note, this appraisal found probiotic-supplemented NAFLD rodents to be presented with improved intestinal barrier function based on reduced serum LPS (*n* = 10/11) and increased tight junction protein (TJ) expressions (n = 9/12) (Figure 3A). This qualitative appraisal identified increased Firmicutes in the distal small intestines and caecum but reduced Firmicutes in the fecal samples. On the contrary, Bacteroidetes were reduced in the caecum and colon but were increased in the feces. The majority of the shortlisted studies reported an increase in lactobacilli and bifdobacteria following probiotic supplementation, suggesting modification of gut microbiota composition by probiotics.

As for the SCFA, this qualitative appraisal denoted mixed effects of caecal butyrate and acetate in probiotic-supplemented rodents. However, increased butyrate was consistently observed in the fecal samples of mice fed with probiotics (37). Lee, Yoon (26) reported on the reduction of fecal endotoxins in probiotic-supplemented NAFLD rodents, which was reflected by the reduced endotoxins production in the gut microbiota. Besides, this qualitative appraisal of probiotic-based *in vivo* studies noted improved intestinal permeability through reduced serum LPS and increased expression of intestinal TJ proteins (Figure 3A) (see Appendix B in Supplementary Material for details of probiotics and finding of each study).

#### Probiotics Reduced HOMA-IR, FBG, and Fasting Insulin of NAFLD *in vivo*

In terms of the glycaemic control parameters, this appraisal found that the probiotic-supplemented NAFLD rodents were presented with reduced HOMA-IR (*n* = 12/18), FBG (*n* = 11/17), and fasting insulin (*n* = 9/13) and/or increased glucagon-like peptide-1 (GLP-1) (*n* = 2/3) (Figure 3A).

#### Probiotics Generally Did Not Alter Body Weight Gain but Lowered the Liver Weight With No Change in Feed Intake of NAFLD-Induced Rodents

Sixty-nine percent of the included probiotic-based studies reported no significant changes were detected on body weight when compared with the control groups (*n* = 27/39) (Figure 3A). The majority of probiotic-supplemented studies were presented with lower liver weight (*n* = 10/10) and liver weight/bodyweight ratio (*n* = 9/14) even though no significant changes were reported in the feed intake (*n* = 9/9) (see Appendix B in Supplementary Material for details of probiotics and findings of each study).

## Discussion

The SYRCLE's RoB tool was used to assess the quality of the shortlisted studies. It comprises 10 items, of which four of them (i.e., sequence generation, grouping, housing arrangement, and random upon assessment) consider the element of randomization. It appears that having investigators or caregivers of animal studies blinded from the interventions, and in this case, probiotics, is not commonly practiced. None of the studies disclosed such practice. Although it is generally understood that random pick of animal should be applied whenever possible, there was no mention of randomization during assessment across the shortlisted studies. Concealment of animal groupings is not a standard practice in animal study (38). Such practice seems impractical as all rodents used in a given study should be of the same species and they generally appear to be identical to one another. As such, selecting any one of the rodents in an assigned group should suffice. Likewise, it is also impractical to blind researchers who are usually the same person who treat the animals. The baseline characteristics are important in an animal study. The age of rodents, for example, is commonly translated into the corresponding human age, and thus, different age reflects different conditions (39). As for the initial body weight at the beginning of the study, it is an important information that can be used to determine as to whether the groups are similar at baseline (20). Animal deaths in the course of the experiment should be clearly indicated as different *n* number of animals described at the beginning of experiment and in the results section would render the data questionable. Blinding of assessor for histology analysis is particularly important as histological evaluation involves judgement of quality.

The improved steatosis as reported by the majority of the shortlisted studies generally involved the use of *Lactobacillus* spp. of various strains either singly (i.e., *Lactobacillus acidophilus, Lactobacillus bulgaricus, Lactobacillus casei, Lactobacillus fermentum, Lactobacillus helveticus Lactobacillus johnsonii, Lactobacillus mali, Lactobacillus paracasei, Lactobacillus plantarum, Lactobacillus reuteri*, and *Lactobacillus rhamnosus*) or in combination (i.e., VSL#3+, Symbiter, VSL#3++++, Poliprobiotic, Probiatop, *Lactobacilli* spp. + *Bifidobacteria* spp. + *Streptococcus thermophilus, L. casei* + *L. helveticus, L. casei* + *L. helveticus, L. casei* + *L. helveticus* + *Pediococcus pentosaceus*, and *L. casei* + *L. helveticus, L. bulgaricus*, six *Lactobacillus* and three *Bifidobacterium*). Nevertheless, there were also shortlisted studies that reported no significant changes in steatosis when *Lactobacillus* spp. were used either singly or in combination (*L. plantarum, L. rhamnosus*, VSL#3+++, VSL#3, mixtures of *L. rhamnosus* + *L. casei* + *L. acidophilus* + *L. plantarum* + *L. fermentum* + *B. lactis* + *B. breve* / *B. bifidum* + *S. thermophilus*. On another note, probiotic strains other than *Lactobacillus* spp. were also reported to reduce steatosis and they included *Akkermansia muciniphila, Bifidobacterium adolescentis, Bifidobacterium animalis, Bifidobacterium longum, Clostridium butyricum, Enterococcus faecalis*, and *P. pentosaceus*. The variations in NAFLD-inducement methods, types of rodents used, and duration of supplementation were amongst the factors that may contribute to the heterogeneity of findings related to steatosis (see Appendix B in Supplementary Material for the details of probiotics used and the findings of each study). Clinically, although liver biopsy and histopathology are not routinely performed for NAFLD monitoring purpose, it remains as the gold standard for definitive clinical diagnosis of NAFLD and confirmation of NASH given the limitations of other non-invasive approaches (40). Liver evaluation using ultrasonography, for instance, is not very sensitive as it detects steatosis only when there is 20% or more fat accumulation in the liver (41). Magnetic resonance spectroscopy, yet another example, is sensitive, but it is costly and not readily available.

Given the asymptomatic nature of NAFLD, its clinical diagnosis is often initiated from an incidental finding of elevated liver enzymes (42). In this systematic review, the liver enzyme that was reduced following probiotic supplementations was mainly ALP. The shortlisted studies that reported reduced serum ALP following probiotic supplementations mainly involved the use of *L. plantarum, Lactobacilli* spp., and/or *B. longum, Bifidobacteria* spp., and *S. thermophilus*. The use of *L. fermentum* and a mixture of 6 *Lactobacillus* + 3 *Bifidobacterium*, however, did not reduce serum ALP (see Appendix A in Supplementary Material for the details of probiotics used and the findings of each study). Since increased serum ALT, in particular, occurs predominantly during NAFL and NASH (43), this parameter is suitable for the measurement of liver injury in both human and NAFLD animal models. In general, liver injury can be assessed based on the levels of liver enzymes in the blood (44), especially the transaminases. This is because the injury of hepatocytes would alter cell membrane permeability, leading to excessive leakage of transaminases like ALT and AST into the circulation. Although the present shortlisted *in vivo* studies reported mixed effects of serum transaminases, close to half of them observed reduced serum ALT following probiotic supplementations, implying their potential hepatoprotection properties against NAFLD-induced liver injury. The mixed effects observed could be attributed to the different duration of probiotic supplementations, induction method, and age during the NAFLD induction. The insignificant findings of serum bilirubin could possibly be attributed to the mixed effects on serum ALT and AST as they both correlated significantly with direct bilirubin, the more soluble form of bilirubin in human serum (45). Owing to bilirubin's possible antioxidant effect, the increment of this parameter has been suggested as a protective biomarker of human NAFLD (45, 46).

The majority of studies that reported on reduced hepatic TAG following probiotic supplementations involved the use of *Lactobacillus* spp. of various strains either singly or in combination. There were three shortlisted studies that used *C. butyricum* of various sub-strains (see Appendix B in Supplementary Material for the details). Excessive intrahepatic TAG is a characteristic of steatosis, the hallmark feature of NAFLD (47). Basically, the main source of the high hepatic TAG pool is the FFA, primarily the serum FFA, which originates from the adipose tissues and dietary fatty acids (48). On the contrary, reduced circulating FFA denotes increased rate of export or catabolism and/or decreased import and synthesis of FFA, which in turn reduces hepatic fat accumulation and lipotoxicity (hepatocyte injury by lipids) (13). Subsequently, these events would initiate a downstream effect on mitochondrial dysfunction, oxidative stress, and ROS production that prevent inflammatory, apoptosis, and fibrogenesis responses, and liver injury (6, 49). Also, reduced hepatic PL is a beneficial effect as it is known as a damaging peroxidative modifier.

As for hepatic cholesterol, the reduced hepatic TC by probiotics would signify an improved cholesterol homeostasis, yielding a balance between cholesterol synthesis, transport, and conversion into bile acids. The bile acids are produced in the pericentral hepatocytes as part of the cholesterol elimination process (50). This appraisal, however, could not draw a general conclusion on bile acids since there was only one shortlisted study (34) that reported changes in bile acid profiles following probiotic supplementation in FXR KO mice. The bile acids function as signaling molecules to the intestines and liver and involve in the regulation of energy homeostasis which could alleviate the severity of NAFLD livers and support hepatic glucose and lipid metabolisms (51).

With regard to serum lipid profile, this qualitative appraisal found improved dyslipidaemia (i.e., reduced hypertriglyceridemia, increased HDL-C levels, and decreased LDL-C particle concentrations) in the majority of the shortlisted studies. Clinically, the state of dyslipidaemia is often measured amongst NAFLD patients to determine the status of their hepatic metabolism function (52). Serum TAG, in particular, is evaluated for the interpretation of liver severity and is used to determine any possible improvement or aggravation of the NAFLD liver (53). This qualitative appraisal also identified increased LDLR in NAFLD rodents supplemented with probiotics (Figure 3A). The LDLR in the liver determines plasma LDL-C levels (54). Basically, the circulating LDL-C is taken up into the liver through LDLR-mediated endocytosis before being metabolized in the liver. This would thus reduce the serum LDL-C (55). The improved hepatic and serum lipid profiles in probiotic-supplemented NAFLD rodents were reflected by the downregulation of the lipogenic pathway. Transcription factors such as ChREBP, SREBP, and PGC-1α control the expression of enzymes that catalyse the rate-limiting steps of liver metabolic processes, thus controlling liver energy metabolism (56). As such, dysfunction of liver signaling would disturb the energy homeostasis, promoting the development of NAFLD through the continuous acquisition of hepatic lipid from circulating FFA (adipose tissue and dietary origins, including up to 30% of dietary sugars) and *de novo* lipogenesis, leading to the accumulation of lipid in the liver (57).

The overall effect of downregulated lipogenic pathway with reduced lipogenic transcription factors (SREBP-1 and ChREBP) resulted in the downregulation of subsequent expression of lipogenic genes. This would reduce the lipogenic enzymes, FAS and ACC and/or increase the fatty acid receptor, PPARα, which in turn improve lipid metabolism through induction of fatty acid oxidation (FAO) or β-oxidation in the liver, increased degradation of fatty acid, regulation of cholesterol transport, and reduction of fat storage (58, 59). Furthermore, increased PGC-1α, a coactivator of PPARα, was also reported in the majority of the shortlisted probiotic-based *in vivo* studies. PGC-1α enhances mitochondrial biogenesis, acts as a molecular switch for liver metabolism, and promotes gluconeogenesis and fatty acid β-oxidation (60). This could explain, at least in part, the significantly reduced hepatic TAG, which may have led to reduced serum TAG as identified in this appraisals. Besides, the increased AMPK phosphorylation and its product, the CPT-1, as reported by majority of the shortlisted studies, signified activation of AMPK. Owing to the role of AMPK as the key regulator for energy homeostasis (61), these outcomes are suggestive of improved glucose uptake, fatty acid β-oxidation, mitochondrial biogenesis, and autophagy and suppressed syntheses of fatty acids and cholesterol, all of which contributed toward the inhibition of lipogenesis (62).

Reduction of MDA implied the potential of probiotics in reducing oxidative stress in NAFLD rodents. MDA is a lipid peroxidation indicator (63), an oxidative biomarker that is cytotoxic and promotes cell death (12). In general, oxidative stress refers to an imbalance between the production of ROS and antioxidant defenses (64). Since oxidative stress contributes, in part, to disease progression of NAFLD, its downregulation would delay the progressive worsening of the disease (12). Furthermore, this qualitative appraisal of the overall oxidative signaling also revealed reduced TGF-β in probiotic-based *in vivo* studies. TGF-β signaling initiates fibrogenic response *via* hepatic stellate cell activation (65). The majority of the shortlisted probiotic-based *in vivo* studies also reported increased Nrf2, a major regulator of cellular redox balance. During oxidative stress, Nrf2 is phosphorylated and activated to interact with antioxidant response element, which will then promote the expression of cytoprotective target genes including the antioxidant enzymes (66). This is consistent with the present qualitative appraisal which denoted increased antioxidant enzymes like SOD and GHS-Px in probiotic-supplemented NAFLD rodents. The increment of these antioxidant enzymes serves as a compensatory regulatory response toward increased oxidative stress (67).

The downregulation of inflammatory cascade was initiated through the reduction of NF-κB, a nuclear transcription factor that regulates various pro-inflammatory cytokines (68), among which TNF-α, IL-6, and IL-1β were reportedly reduced. Besides, the majority of the shortlisted probiotic-based *in vivo* studies also reported increased anti-inflammatory cytokines like IL-10, of which their production can be induced by the presence of IL-4 (69). In addition, NAFLD rodents supplemented with probiotics were presented with reduced MCP-1, a chemokine with pivotal roles in the development of inflammatory responses and crucial for immune cell recruitment to inflammation sites (70), both of which contribute to the progression of hepatic inflammation and fibrosis (71). Taken together, these findings implied the potential of probiotic supplementations in halting NAFLD progression in rodents through the suppression of inflammatory pathway and prevention of fibrosis (72). In addition, gut microbiota-derived endotoxins are also pro-inflammatory in nature. Its reduction in probiotic-supplemented NAFLD rats would help to suppress the inflammatory cascade.

The level of gut microbiota-derived endotoxins also reflects dysbiosis of the host (73). Therefore, the reduction of endotoxins levels in the probiotic groups is suggestive of improved gut microbiota balance. A previous clinical study reported that human NAFLD was associated with dysbiosis, independent of body mass index and insulin resistance (74). On the contrary, a preclinical study found germ-free mice to be relatively more resistant to HFD-induced hepatic lipid accumulation when compared with conventional mice, demonstrating the role of gut microbiota in NAFLD development (75). These data implied that altered gut microbiota may play a causal role in the development of NAFLD, rather than a mere consequence of it. To date, the specific bacterial species that may have caused NAFLD and their molecular crosstalk with the host during pathogenesis remain elusive (73). Nevertheless, it was found that NAFLD patients harbored more Gram-negative bacteria than Gram-positive bacteria when compared with healthy individuals (76). Gram-negative bacteria belonging to the family *Enterobacteriaceae* produces proinflammatory endotoxin, while at genus level, *Enterobacter* is an endotoxin-producing pathogen (77). Together with LPS, the endotoxin-producing gut microbiota could be the causative agents for NAFLD owing to the upstream effects on inflammatory pathway (73). Elsewhere, it was reported that NASH patients were presented with increased Bacteroidetes and reduced Firmicutes phyla when compared with healthy subjects (78). At the genus level, stool samples of NASH patients showed lower proportions of *Prevotella* spp. and higher proportions of *Bacteroides* spp. (15). In a 16-week-HFD-induced C57BL/6J mice, interindividual differences (i.e., responder or non-responder-receivers) were reported, in which transplantation of their gut microbiota into germ-free mice (fed with HFD for another 16 weeks) revealed aggravation of steatosis, glycaemia, and insulin resistance in responder-receiver mice (79). It appears that intestinal microbiome modulates the metabolic and hepatic consequences of the diet consumed.

Generally, the SCFA is an important energy source and plays an important role in the regulation of physiological function which is vital for the intestinal epithelial cells (76). The types of SCFA and amount produced may vary depending on the gut microbiota composition and the amount of carbohydrate consumed (5). The main SCFA produced by gut microbiota includes acetate, propionate, and butyrate (80). The importance of Bacteroidetes and Firmicutes lies in their contribution to the SCFA production. The Bacteroidetes phylum mainly produced acetate and propionate as its metabolic end products, while Firmicutes mainly produce butyrate (81). Interestingly, increased butyrate seems to yield anti-inflammatory effect (37). Furthermore, Endo, Niioka (82) demonstrated that butyrate inhibited NAFLD progression through the activation of AMPK signaling pathway.

Not all studies that investigated the effects of probiotic supplementations on gut microbiota in NAFLD investigated the level of endotoxin or LPS and SCFA production. In fact, there was only one shortlisted study by Wang, Xu (35) investigated the effects of probiotic supplements on changes of intestinal gut microbiota, serum LPS, and caecal SCFA. Wang, Xu (35) reported that supplementation of probiotics in HFD-induced C57BL/6N mice with *L. plantarum* X (1 × 10^8^ CFU/ml) + *B. bifidum* V (2 × 10^8^ CFU/ml) mixture for 6 weeks presented with increased Bacteroidetes/Firmicutes ratio, increased Bacteroides, Lactobacillus, and Parabacteroides; reduced serum LPS; and increased butyric and acetic acids (data available at no 40, Appendix B in Supplementary Material). The majority of the shortlisted studies that investigated both gut microbiota and endotoxin or LPS reported changes of gut microbiota composition which were accompanied by reduced serum or portal blood LPS in the NAFLD-induced mice following supplementations of *Lactobacillus* spp. of various strains, either singly (*L. rhamnosus, L. johnsonii, L. paracasei*, L. casei, *L. bulgaricus, L. helveticus*) or in combination (*L. plantarum* + *B. bifidum*) (data available in Appendix B in Supplementary Material). There were, however, three shortlisted studies that reported no change in gut microbiota and stool endotoxins despite being supplemented with probiotics containing multiple strains of *Lactobacillus* (data available at no 43, Appendix B in Supplementary Material). On another note, supplementation with *P. pentosaceus* altered the gut microbiota composition and reduced stool endotoxin. Elsewhere, it seems that higher concentration of *L. paracasei* is required to produce the desired effects on gut microbiota and LPS as concentrations lower than 4 × 10^10^ cfu reported no change (data available at no 39, Appendix B in Supplementary Material). The majority of the shortlisted studies that investigated SCFA production reported increased SCFA following supplementation with *Lactobacillus* spp.

The increased expression of intestinal TJ proteins (i.e., ZO-1, ZO-2, occluding, and claudin) is an indicator of improved gut barrier function, in which the entry of substances through the paracellular pathway of the intestinal mucosa would be better controlled, reducing the entry of insults, particularly the LPS (83). Alternatively, the gut barrier function *in vivo* is often assessed using oral administration of sugar of known molecular weight and subsequent direct measurement of the sugar level in the blood (e.g., 4,000 Da dextran) or urine after a duration of time (e.g., lactulose/ mannitol ratio), or *via* indirect analysis of intestinal TJ gene expression [e.g., toll-like receptor 4 (TLR4)] (84, 85).

The mechanism of insulin resistance is related to energy metabolism, particularly in the liver. In NAFLD, increased hepatic TAG would lead to steatosis and subsequently hyperinsulinaemia, thus giving rise to the insulin resistance state (47). As a result, the insulin-mediated suppression of glucose production would be impaired and the total endogenous glucose production increased (13, 86). Probiotic supplementations, however, improved glycaemic parameters by reducing HOMA-IR, FBG, and fasting insulin, all of which are positive routes toward hepatoprotection against insulin resistance which plays important role in the pathogenesis of NAFLD (13). On another note, the GLP-1 receptor is downregulated in NAFLD, and GLP-1 receptor agonist has been suggested as a potential therapeutic (87). The main physiological role of GLP-1 is to maintain glucose homeostasis by promoting insulin release and reducing glucagon secretion, postprandially. The upregulation of GLP-1 induced by probiotic supplementations could be crucial against NAFLD.

The reduced organ weight despite of no changes detected in the feed intake could be attributed to the underlying effect of adipocyte-derived hormones, leptin, and adiponectin, which exhibited mixed effects. Leptin is a satiety hormone that inhibits food intake (88), while adiponectin stimulates food intake (89). As such, the adiponectin/leptin signaling is essential in regulating food intake. Given that adipose tissue is the source for FFA to the liver, reduction of adiposity should contribute to the reduced circulating FFA. However, mixed effects on adiposity [Lee index, an obesity index for rodents (90)] by probiotics *in vivo* were based on the findings reported by Savcheniuk et al. (91) and Mohammed et al. (92) (see Appendix B in Supplementary Material). More studies are required before a conclusion for probiotics' effects on adiposity can be drawn.

**Figure 4:**
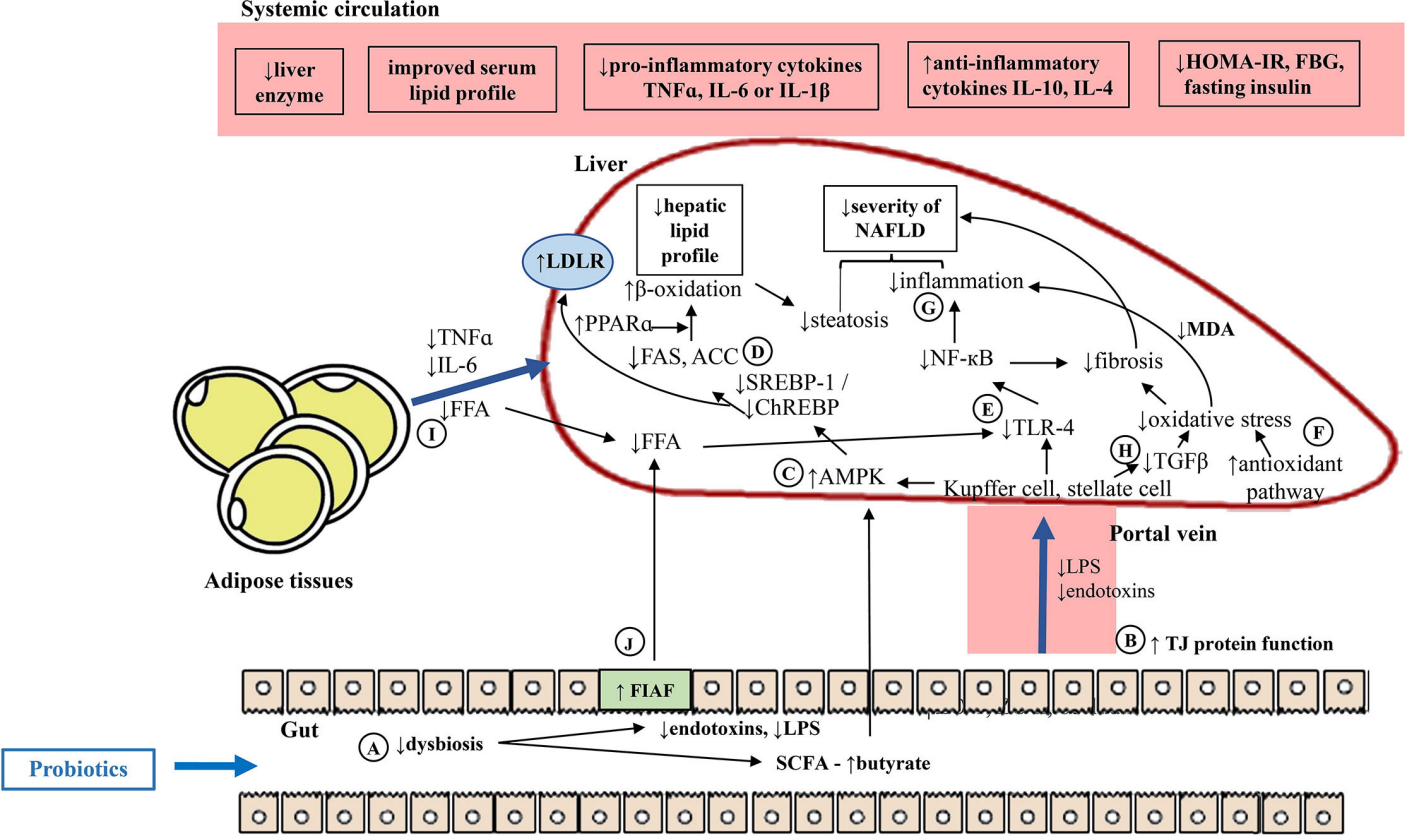
Potential mechanisms underlying probiotic-mediated prevention of NAFLD *in vivo*. **(A)** Probiotic-based interventions could improve dysbiosis, and in turn modulate the gut microbiota-derived metabolites. **(B)** Concurrently, improved gut barrier function would limit the delivery of gut-derived insults. **(C)** In the liver, the increased butyrate would increase activation of hepatic AMPK. **(D)** This would downregulate the hepatic lipogenesis pathway through reduced transcription factors and lipogenic enzymes and/ or increased PPARα, improved fatty acid β-oxidation. **(E)** The reduced microbiota-derived insults to the liver, would reduce the activation of TLR-4, downregulating the oxidative and inflammatory responses. **(F)** The reduced hepatic MDA and increased antioxidants mechanisms, and/ or **(G)** reduced NF-κB would prevent further oxidative and inflammatory stresses to the liver. **(H)** Lesser liver injury would reduce the stellate cell activation, reducing the TGF-β and downregulate the fibrogenic pathway. **(I)** Improved hepatic lipid metabolism could be seen through improved dyslipidaemia, reduced adiposity, FFA supply and secretion of pro-inflammatory adipokines. **(J)** Increased butyrate would promote FIAF, reducing FFA source to the liver. ACC, Acetyl-CoA carboxylase; AMPK, AMP-activated protein kinase; ChREBP, carbohydrate response-element binding protein; FAS, fatty acid synthase; FBG, fasting blood glucose; FFA, free fatty acid; FIAF, fasting-induced adipose factor; GLP-1, glucagon-like peptide-1; HOMA-IR, homeostasis model assessment for insulin resistance; IL-1β, interleukin-1; IL-4, interleukin-4; IL-6, interleukin-6; IL-10, interleukin-10; LDLR, low-density lipoprotein receptor; LPS, lipopolysaccharide; MDA, malanoyl dialdehyde; NF-κB, necrosis factor kappa B; PPARκ, peroxisome proliferator-activated receptor alpha; SCFA, short chain fatty acid; SREBP-1, sterol regulatory element-binding protein 1; TGF-β, transforming growth factor beta; TJ, tight junction protein; TLR4, toll-like receptor 4; TNF-α, tumor necrosis factor alpha; ↑, increased; ↓, reduced.

Figure 4 shows the overview of potential mechanisms underlying probiotic-driven prevention of NAFLD *in vivo*. Supplementation of NAFLD rodents with probiotics improved the body energy homeostasis through the gut-liver-adipose axis by regulating lipid and/or glucose metabolisms *via* the lipogenic pathway and the oxidative and/or inflammatory pathways. Probiotic-based interventions could bring about positive changes to the gut, particularly correcting dysbiosis. This was evident by the increased *Lactobacillus* spp. which could in turn change the production of gut microbiota-derived metabolites (i.e., reduced endotoxins and LPS but increased SCFA production, particularly butyrate) (Figure 4A). Concurrently, the increased expression of intestinal TJ proteins would regulate the paracellular permeability by limiting the delivery of endotoxins and LPS or other gut microbiota-derived metabolites from the gut, protecting the liver from inflammatory insults given that the liver is the first organ to receive the majority of blood drained from the intestines (Figure 4B). Activation of hepatic AMPK, the energy sensor for energy metabolism, would ensue to downregulate the lipogenic pathway (Figure 4C) through the reduction of transcription factors (i.e., SREBP-1 or ChREBP) and/or reduction of lipogenic enzymes (i.e., FAS and/ or ACC) but with the increment of PPARα and restoration of β-oxidation capability of the liver (Figure 4D). This would, therefore, reduce the hepatic lipid profile (i.e., reduced hepatic TAG), which may be partly regulated by bile acid metabolism, leading to reduced steatosis. In the liver, the diminished presence of LPS would reduce the activation of the TLR4-dependent NF-κB inflammatory pathway, leading to the downregulation of inflammatory responses and injury (Figure 4E). At the same time, the reduced hepatic MDA and increased antioxidants mechanisms would suppress the oxidative pathway, protecting the liver from inflammation (Figure 4F). The reduced number of injured hepatocytes would release lesser damage-associated molecular pattern molecules (DAMPs) and therefore reduce the subsequent inflammatory response and recruitment of immune cells (Figure 4G). The reduced activation of hepatic stellate cells would also prevent the progression of NAFLD through downregulation of the fibrogenic pathway (Figure 4H). On the contrary, the reduced adiposity would signify reduced secretion of adipokines, TNF-α and IL-6, reducing the inflammatory insults (Figure 4I). On another note, butyrate is capable of stimulating the secretion of fasting-induced adipose factor (FIAF), also known as angiopoietin-like protein-4 from intestinal epithelium, which reduces lipoprotein lipase (LPL) that releases triglycerides from circulating chylomicrons and VLDL, thus reducing the FFA source to the liver. In addition, reduced FFA also would reduce the activation of TLR4 (5) and its downstream effects, preventing the progression of the disease (Figure 4J).

## Conclusion and Future Perspective

This systematic review implied the preventive potentials of hepatoprotective probiotic supplementations against NAFLD *in vivo*. Probiotic supplementations against NAFLD *in vivo* appear to alleviate the disease presentation itself through marked improvements of liver histopathology. The evident improvement of metabolic functions of NAFLD probiotic supplemented rodents was also suggestive of the alleviation of NAFLD-related comorbidities like dyslipidaemia. The ability of probiotic supplementations in modulating the gut microbiota composition appears to be the driving factor for hepatoprotection mediated through reduction of gut microbiota-derived endotoxins production, downregulation of lipogenic, and oxidative and inflammatory pathways with/without improved gut barrier function. That having said, the limitations of existing findings raise the need for further in-depth studies to further understand the beneficial effects of probiotics against NAFLD. As far as the gut-liver-adipose axis is concerned, although adipose tissue dysfunction was recognized as part of NAFLD pathology, there remains limited studies on hepatoprotection by probiotics in terms of the molecular aspects of adipose tissue. Besides, there is also gap in knowledge on therapeutic effects of probiotics against NAFLD through modulation of gastrointestinal motility. As much as the autonomic nervous system is known to innervate the liver and play a role in energy homeostasis, the effect of probiotic supplementations on neuroendocrine and neurotransmitter and their neuro-modulatory effect in the context of NAFLD have yet to be elucidated.

## Data Availability Statement

The original contributions presented in the study are included in the article/Supplementary Material, further inquiries can be directed to the corresponding author.

## Author Contributions

FS, SML, CFN, and KR independently searched, extracted, assessed all the potential studies, and assessed the eligibility and risk of bias of all potential studies identified for inclusion. FS drafted the manuscript and conducted the data analyses. KR, SML, and CFN reviewed and edited the manuscript. All authors approved the final version of the manuscript.

## Funding

This work was supported by the Ministry of Education Malaysia under Fundamental Research Grant Scheme [600-IRMI/FRGS 5/3 (317/2019)].

## Conflict of Interest

The authors declare that the research was conducted in the absence of any commercial or financial relationships that could be construed as a potential conflict of interest.

## Publisher's Note

All claims expressed in this article are solely those of the authors and do not necessarily represent those of their affiliated organizations, or those of the publisher, the editors and the reviewers. Any product that may be evaluated in this article, or claim that may be made by its manufacturer, is not guaranteed or endorsed by the publisher.
