# Supplementary material for: Hepatoprotection of Probiotics Against Non-Alcoholic Fatty Liver Disease in vivo: A Systematic Review
Source: Front Nutr. 2022 Apr 11;9:844374. doi: 10.3389/fnut.2022.844374 (PMC9035816; doi:10.3389/fnut.2022.844374)
Supplement: Supplementary file 1 [file Data_Sheet_1.docx]

**Appendix A: Criteria for risk of bias assessment based on the SYRCLE’s RoB Tool.**

(Table continued overleaf)

| **Criteria*** | **Low** | **Unclear** | **High** |
| --- | --- | --- | --- |
| **Selection bias** | | | |
| 1. Sequence generation | Description of method used to generate sequence of allocation, for randomisation purpose. | Mentioned randomised but no details or no information on randomisation.  e.g.: The authors neither described “*the randomisation approach applied (if any)*” nor explained as to whether “*the groupings were performed using proper randomisation techniques*” (such as usage of computer random number generator). | Inappropriate method of randomisation (e.g.: by birthdate) |
| 1. Baseline | Baseline characteristics stated (i.e. gender, age, body weight and equal time of inducement). | Not applicable. | Any one of the baseline characteristics not stated. |
| 1. Grouping | Evidence of adequate concealment of groups. | No explanation on assignment of groups. | Allocation allow foresee assignments of groups. |
| **Performance bias** | | | |
| 1. Housing arrangement | All animals were housed in the same house and environment (identical housing condition, with controlled environment). | Unknown housing arrangement. | Different housing arrangement between groups (non-identical housing condition). |
| 1. Blinded researcher | Evidence of caregiver blinded to interventions. | Blinded but no details on how it was conducted.  e.g.: There was no evidence of either “researchers being blinded for administration of interventions” (i.e., pro-, pre- or synbiotic). | Caregiver was not blinded to the interventions. |
| **Detection bias** | | | |
| 1. Randomisation upon assessment | Evidence of random pick of animal during assessment  e.g.: random table or computer-generated number). | Randomly picked for assessment but no explanation on how it was conducted.  e.g.: There was no evidence on “the use of random component during assessment” (such as reference to a random number table). | No information on random pick of animal during assessment. |
| 1. Blinded histology assessor | Evidence of assessor blinded. | Histological analysis conducted but no mention of blinding. | No histological analysis conducted. |
| **Attrition bias** | | | |
| Attrition bias | All animals were included in analysis. Any missing of animal data was explained. | Number of animals was not stated clearly  e.g.: n/group was mentioned only in the materials and methods section but the ultimate n/group was not stated in the results section. | No explanation on missing animal data  e.g.: starting n in the materials and methods section > ultimate n in the results section. |
| **Reporting bias** | | | |
| Reporting bias | Free of selective reporting based on methods and results. All expected outcomes were included. | Insufficient data to determine reporting bias. | Selective reporting  e.g.: a research objective was mentioned but no corresponding results reported. |
| **Other potential bias** | | | |
| Presence of other potential risk of bias | Free of other risk of bias. | Insufficient data to determine other risk of bias. | Editorial error in the published paper that may mislead reader.  e.g.: discrepancies of findings in figure, supplementary data and text that could be potentially misleading. |

* The definition of each criterion was refined based on the context of the present study.
