# Supplementary material for: Hepatoprotection of Probiotics Against Non-Alcoholic Fatty Liver Disease in vivo: A Systematic Review
Source: Front Nutr. 2022 Apr 11;9:844374. doi: 10.3389/fnut.2022.844374 (PMC9035816; doi:10.3389/fnut.2022.844374)
Supplement: Supplementary file 2 [file Data_Sheet_2.docx]

**Appendix B: Summary of findings from all studies included for the present systematic review (n = 44).**

Table continued overleaf

| **No.** | **Author** | **Animal model** | **Inducement** | **Microbial intervention** | **Duration**  **(week)** | **Liver histology** | **Liver enzyme** | **Body / organ weight** | **Metabolic energy metabolisms**  **(Glucose / lipid metabolism)** | **Inflammatory / Oxidative pathway** | **Gut microbiota / intestinal permeability** | **Others** |
| --- | --- | --- | --- | --- | --- | --- | --- | --- | --- | --- | --- | --- |
| **No.** | **Studies included in the systematic review** | | | | | | | | | | | |
| 1 | Ma, Hua (1) | C57BL6 mice  Male  6-8 wo  n=3/group | **†**HFD (60% fat)  8 wks | **PRO:** VSL#3+  (1.5 × 10^9^ colonies/mouse/ day)  gavage | 4 | ↓S  (scoring: Li, Yang (2)) | - | ↓ BW | ↓ hepatic TAG  ↓ IR | ↓ expressions of hepatic TNF-ɑ  ↑ IL-4  ↓IKK-ꞵ activity  ↓NF-кB binding | - | ↓ hepatic NKT cell depletion |
| 2 | Velayudham, Dolganiuc (3) | C57BL/6 mice  Female  6 wo  n=6/group | **†**MCD  10 wks | **PRO:** VSL#3+++  in drinking water | 9 | ↔S, ↓F  (scoring: own) | ↑ALT | ↔ Liver/BW ratio | ↑ hepatic TAG  ↑ hepatic PPARα, PPARγ (mRNA)  ↑ PGC-1ɑ (protein) | ↑ hepatic TNF-α | Gut permeability:  ↑ serum endotoxin  ↑ hepatic TLR4, CD14, MD-2 & MyD88 | ↓ hepatic procollagen I-  1ɑ (mRNA)  ↓ MMP-2 & MMP-9 |
| 3 | Bhathena, Martoni (4) | Bio F1B Golden Syrian hamster  Male  8 wo  90g  n=12/group | **†**MCD  5 wks | **PRO:** *L. fermentum* ATCC 11976  11.51 log cfu/mL (microencapsulate)  gavage | 12 | ↓S, total hepatic lipid  (scoring: own) | ↓ALT  ↓GGT  ↔AST  ↔ALP | ↓ gross liver wt | ↓ hepatic TAG, FC, EC, PL  ↓ serum TAG, TC, EC, HDL-C, NEFA  ↔ serum FC  ↓ HMG-CoA reductase & HMG-CoA reductase:CYP7A1 ratio  ↓ fasting serum insulin, ↔ FBG  ↓ HOMA-IR | - | - | - |
| 4 | Savcheniuk, Kobyliak (5) | Wistar rats  Male & Female new-born  n=10/group | **†**MSG-induced (1st 4-mo on ND) MSG (4.0 mg/g BW) s.c. on post-natal day-2,4,6, 8 and 10 | **PRO:** Symbiter  140 mg/kg (1.4 × 10^10^ CFU/kg)  gavage | 12 | ↓S, ↓I  (scoring: Kleiner, Brunt (6)) | - | ↓ BW  ↓ visceral AT  ↓ obesity  (Lee index) | ↑ adiponectin  ↓ visceral AT leptin  ↓ FBG, insulin & HOMA-IR | - | - | - |
| 5 | Liang, Webb (7) | C57BL/6 mice  Male  Adult  n=5/group | **†**HFD (60% fat)  8wks | a. **PRO:** VSL#3++++  (1.5 x 10^9^ colony/mice/day)  b. **PRO:** VSL#3++++  (1.5 x 10^8^ colony/mice/day)  c. **PRO:** *B. infantis* | 4 | ↓S (a. only)  (scoring: Ma, Hua (1)) | - | ↓ BW (a. only) | ↓ hepatic TAG (a. only)  ↓ fasting insulin (a. and c. only)  ↓ HOMA-IR (a. only) | - | TLR4 knockout showed no protection against HFD-induced hepatic NKT depletion | ↑ hepatic NKT cell depletion (a. only) |
| 6 | Ivanovic, Minic (8) | C57BL/6 mice Male  6–8 wo  n=10/group | **†**HFD (21% fat and 20% protein)  4 wks | a. **PRO:** *L. plantarum* WCFS1  (2 × 10^9^ cfu)  b. **PRO:** *L. rhamnosus* LA68 2 × 10^9^ cfu  gavage | 12 | ↔S  (scoring: Kleiner, Brunt (6)) | ↔ALT  ↔AST | ↓ BW gain  ↔ food intake | ↓ serum TAG, LDL-C (a. only) ↓ serum TC, HDL-C (b. only)  ↑ serum leptin (a. only)  ↓ adiponectin (b. only)  ↔ GTT | ↓ serum TNF-α  ↓ serum IL-6  (a. only) | - | ↑CD3+CD8+ cell count, ↓CD19+ cells,  ↓ CD25+ cell count (a. only) |
| 7 | Briskey, Heritage (9) | C57B1/6J background mice  Male  8 wo  n=9-10/group | **†**HFD 23% total fat (10wks) | **PRO:** *L. rhamnosus* / *L. casei* / *L. acidophilus*/ *L. plantarum* / *L. fermentum* (82%) & *B. lactis* / *B. breve* / *B. bifidum* (13%) & *S. thermophilus* (5%)  in drinking water | 10 | ↔S, ↔I  (scoring: Kleiner, Brunt (6)) | ↔ALT ↔AST | ↔BW | ↓ hepatic TAG ↔ serum TAG, TC, albumin  ↔ glucose | - | Gut permeability:  ↑ ZO-1, ZO-2 expressions  (large intestine) | - |
| 8 | Kobyliak, Falalyeyeva (10) | Wistar rats  Male  new-born  n=10/group | **†**MSG-induced NAFLD (4.0 mg/g of BW) (s.c. injection on post-natal day- 2, 4, 6, 8 and 10) | a. **PRO:** *B. animalis* VKL b. **PRO:** *B. animalis* VKB c. **PRO:** *L. casei* IMVB-7280 d. **PRO:** Poliprobiotic (mixture of a., b. & c.)  e. **PRO:** Symbiter  gavage | 12 | ↓S, ↓I  (scoring: Kleiner, Brunt (6)) | ↔ALT ↔AST | ↔ BW | ↓hepatic total lipid & TAG  (d. and e. only)  ↔ total bilirubin, indirect bilirubin, direct bilirubin | - | - | - |
| 9 | Kim, Park (11) | C57BL/6J mice  Male  4 wo | **†**HFD (60% fat)  2wks | **PRO:** *L. rhamnosu*s GG  (1x10^8^ cfu per mouse)  orally | 13 | ↓S  (scoring: own) | - | ↔ BW  ↔ calorie intake  ↓ liver wt  ↓ mesenteric & s.c. AT | ↓ serum TAG & TC  ↓ PPARγ & SREBP1c (mRNA)  ↓ CD36, ApoB100,  ↓ SREBP2, HMGCR, HMGCS  ↓ LDLR, SR-B1  ↓ ApoB100  ↑ ABCG5, CYP7A1  ↓ FXR, SHP  ↓ FGFR4, β-Klotho | ↓ IL-6 & IL-12 (mRNA)  ↔ IL-10 | GM of stool:  ↔ Firmicutes:Bacteroidetes ratio |  |
| 10 | Zhou, Pan (12) | C57BL/6 mice  Male  n=10/group | **†**HFD (88% standard diet, 10% lard and 2% cholesterol)  8wks | **PRO:** *C. butyricum* B1 (1 x 10^9^ cells)  gavage | 8 | ↓S, ↓F  (scoring: Kleiner, Brunt (6)) | ↓ ALT ↓AST | ↓ BW  ↓ liver index  ↓ epididymal AT index | ↓ hepatic TAG and TC  ↑ hepatic PPAR-γ (mRNA) ↓ hepatic PPAR-α (RNA) ↑ EAT PPAR-α & PPAR-γ (mRNA)  ↓ FBG, HOMA-IR and ISI | ↓TNF-α, MCP-1, IL-1β, IL-2, IL-6 &  IL-10 (mRNA)  ↓ EAT TNF-α, MCP-1  ↑ hepatic & ileal Foxp3, IL-4 & IL-22  ↓ IFN-γ & IL-17 | ↓ TLR4 & Myd88 mRNA | Hepatic & caecal SCFAs:  ↑ butyric acid  ↔ acetic &  propanoic acids |
| 11 | Jena, Sheng (13) | C57BL/6  FXR KO mice Male  12 wo  n=6-10/group | **†**Western Diet (21.2% fat, 34% sucrose & 0.2% cholesterol)  36 wks | a. **PRO:** *B. longum* subsp. infantis 10^9^ CFU/mouse  gavage | 8 & 28 | ↓S  (scoring: own) | ↓ ALT  ↓ ALP | - | ↓ hepatic TAG and TC  Change hepatic and serum bile acid profile  ↓ blood glucose post insulin injection | ↓ hepatic IL-1β, IL-6, TNF-α, Timp1, MCP-1, COL1A1, CCL17, and CCL20 expressions  (by 8wks)  ↓ IL-1β, IL-6, TNF-α, TIMP1, MCP-1 (↓ileal inflammatory genes) ↑ ileal IL-10 and Reg3γ | ↔ abundance of caecal bacterial gene baiJ, (responsible for secondary BA synthesis) | ↔ Inos  ↓ hepatic lymphocyte  ↓ hepatic and illeal genes Il1β, Il6, Ccl17, Ccl20, Cxcl10 and ifnγ |
| 12 | Kobyliak, Abenavoli (14) | Wistar rats  Male  new-born  n=10/group | **†**MSG-induced NAFLD (4.0 mg/g of BW) (s.c. injection on post-natal day- 2, 4, 6, 8 and 10) | **PRO:** Symbiter 2.5 mL/kg  gavage | 12 | ↓S, ↓I, ↓B  (scoring: Kleiner, Brunt (6)) | ↔ALT ↔AST | - | ↓ hepatic total lipid & TAG  ↔ total bilirubin, indirect bilirubin, direct bilirubin | ↓IL-12, ↑ IL-4 & TGF-ꞵ  ↔IL-1ꞵ, IL-10, IL-12B p40, IFNɣ | - | - |
| 13 | dos Santos, Ribeiro (15) | Wistar rats  Male  8-9 wo  ~350g  n=5-10/group | **†**AIN-93M+1% cholesterol&0.35% cholic acid  (30days) | **PRO:** Probiatop®  gavage | 30days | ↓S  (scoring: own) | ↓ALT  ↓AST | ↔ BW | - | ↑ NFкB  ↔ TLR-4 &  ↔ TNFɑ (mRNAs) | - | - |
| 14 | Zhao, Liu (16) | FGF21 knockout (KO) and  C57BL/6 (WT) mice  Female  6-8 wo  n=5-8/group | **†**30% fructose-containing water  5 or 12 wks | **PRO:** *L. rhamnosus* GG 10^9^ CFU/day  gavage | 4 | ↓S  (scoring: Wang, Kirpich (17)) | ↔ ALT  ↔ AST | ↓ gWAT wt and adipocyte size in WAT (WT only)  ↑ serum adiponectin, ↔hepatic adiponectin  ↑hepatic CEB/Pb  (WT only) | ↓ hepatic TAG, TC  ↓ serum TAG (WT only)  ↔ serum LDL-C, HDL-C & VLDL  (WT only)  ↓ FAS & SCD1 (WT only)  ↓ total hepatic ChREBP  & prevented ChREBP nuclear translocation (WT only)  ↑ serum FGF21  ↑CPT1 (WT only) | ↓ hepatic TNF-α, CXCL10 (mRNA)  (WT only) | - | ↓apoptotic cell  (WT only) |
| 15 | Mohammed, Magdy (18) | Wistar rats  Male  150g  n=10/group | **†**HFD  10wks | **PRO:** *L. plantarum* EMCC-1039 (1.2 x 10^9^ cfu/ml)  gavage | 2 | ↓S, ↓I, ↓F  (scoring: Kleiner, Brunt (6)) | ↓ALT  ↓AST | ↓ BW % change  ↔ Lee index | ↓serum TAG, TC, HDL-C and  LDL-C | - | ↓hepatic TLR4 and BDNF | - |
| 16 | Park, Lee (19) | Wistar rats  Male  n=8/group | **†** HFD/F (45% fat and 10% fructose in drinking water)  8wks | a. **PRO:** *L.* *plantarum* ATG-K2  (5 × 10^8^ cfu)  b. **PRO:** *L.* *plantarum* ATG-K6  (5 × 10^8^ cfu)  gavage | 8 | ↓S  (scoring: own) | ↓ALT  ↓AST  ↓ALP | ↓ BW gain | ↓ hepatic TAG, TC  ↓ serum TAG, TC and leptin  ↑ HDL-C, serum adiponectin  ↓ hepatic SREBP 1c, FAS (mRNA)  ↑ hepatic pAMPK, pAMPK/ACC and CPT1 (K2 only)  ↓ FBG  Table continued overleaf | ↓ serum SOD (b. only), GPx and CAT  ↓ hepatic MDA | GM of stool:  67.2%–77.1% abundance of Firmicutes and 12.4%–25.8% abundance of Bacteroidetes | - |
| 17 | Rashid, Khan (20) | Albino rats  Male  4 wo  n=6/group | **†**HFHS  (High fat high sugar group)  (36%) and high sugar (40%)  14 wks | **PRO:** *Lactobacilli* spp., *Bifidobacteria* spp. and *S. thermophilus* 2 x 10^6^ CFU/gram  method of administration not mentioned | 4 | ↓S  (scoring: Brown and Kleiner  2016) | ↔ ALT  ↓ AST  ↓ ALP | - | ↓ serum TAG, TC, total protein and globulin  ↑ serum HDL-C,  ↔ serum LDL-C, albumin & albumin:globulin ratio | ↓ serum TOS and MDA  ↑ serum TAC  serum SOD | ↓ damaged villi, illial cytoplasmic vacuolation and thickened intestinal muscle layer | alleviate renal damage (↓ cytoplasmic vacuolation, tubular necrosis, tubular thickening, interstitial cell infiltration) |
| 18 | Li, Yang (2) | C57BL-6 (ob/ob) mice  Male  n=12-18/group | - | **PRO:** VSL#3 (1.5 x 10^9^ colonies/  mouse/day)  added to fresh liquid diet | 4 | ↔S, ↓I  (scoring: own) | ↓ALT | - | ↔ hepatic total fatty acid content  ↓ oleic acid  ↓ fatty acid ꞵ oxidation,  ↓ hepatic FA, UCP-2 (mRNA) | ↔ hepatic TNFɑ (mRNA)  inhibit JNK &  ↓NF-кB binding | - | - |
| 19 | Esposito, Iacono (21) | Sprague Dawley rats  Male  at weaning  113.06±2.5g  n=6/group | HFD (71%) | **PRO:** VSL#3++  gavage | 4 | - | ↔ALT  ↓AST | ↔ BW gain  ↓ fat mass & liver wt  ↔ food intake | ↓ serum TAG, LCPT I  ↑ PPARα  ↔serum insulin | ↓ hepatic MDA  ↓ hepatic TNF-α, COX-2, NF-кB  ↓ pro- and active MMP-2 and proMMP-9 activities | - | ↓protein nitrosylation and iNOS |
| 20 | Karahan, Isler (22) | Wistar rat  Male  n=6/group | MCD | a. **PRO:** *L. fermentum* (BB16-75, AK2-8, AK5-22, AK6-26), *L. plantarum* (AA17-73, AK7-28, AK8-31B) and *E. faecium* (AB6-21, AB16-68,AK4-120, AK7-31, BK9-40, BK13-54)  b. **PRO:** *E. faecium* BK10-47 and *L. plantarum* (AB7-35, AC3-16, AC21-101, AB16-65, BK10-48)  gavage | 2-6 | ↓S & ↓ I  (a. only)  (scoring: Sundaram, Whitington (23)) | ↔ALT | - | - | ↓TNF-α expression  (a. only) | - | ↓ apoptotic markers:  Bax, caspase 3, caspase 8, and Bcl-2 (a. only) |
| 21 | Xu, Wan (24) | Sprague Dawley rats  Male  150–180g  n=10/group | HFD (60% fat) | a. **PRO:** *L. acidophilus*  (CGMCC 2106)  b. **PRO:** *B. Iongum*  (CGMCC 2107)  in drinking water (10^10^/mL) | 12 | ↓S  (scoring: own) | ↔ALT | ↔ BW | ↓ hepatic lipid (b. only)  ↔ serum TAG, TC | - | GM of stool: ↑Bifidobacterium  Intestinal permeability:  ↔ Lactulose/Mannitol ratio | - |
| 22 | Endo, Niioka (25) | Fischer 344 rats  Male  70g  n≥6/group | CDAA | **PRO:** *C. butyricum* MIYAIRI 588  8.5 x10^9^ cfu/g  mix in food | 2-50 | ↓S, ↓F  (scoring: own) | ↓ALT | ↓ BW | ↓ hepatic TAG  ↓ hepatic AMPK phosphorylation, SREBP-1c, UCP2 & PPAR-γ  ↔ levels of bile salt export pump  ↓ fasting insulin, HOMA-IR but  ↔ FBG | ↓ hepatic NF-kB & TNF-α  ↓ hepatic 4-HNE & MDA  ↓ hepatic Nrf2 | Gut permeability:  ↑ ZO-1 & occludin of intestinal tissue (proteins)  ↓ endotoxin  (portal vein) | - |
| 23 | Okubo, Sakoda (26) | C57BL/6 mice  6 wo  n=12-18/group | MCD | **PRO:** *L. casei* Shirota  10^9^ cfu/mouse/day  gavage | 6 | ↓S, ↓F  (scoring: own) | ↓ALT | ↔ BW | ↓ hepatic TAG, SREBP-1c, FAS | ↓ hepatic TNFα, nuclear NFкBp65-positive cells in colon (IS) | GM of faeces:  ↑ Bifidobacterium  ↓*Clostridium cocoides*  Gut permeability:  ↓ serum LPS | Suppressed fibrosis |
| 24 | Seo, Inoue (27) | Sprague Dawley rats  Male  5 wo  n=6/group | HFD (60% fat) | **PRO:** *C. butyricum* MIYAIRI 588 (CBM588) 1.4 x 10^9^ cfu/day  HFD coated with CBM588 spores | 12 | ↓S  (scoring: own) | - | ↔ BW  ↔ feed intake | ↓ hepatic TAG, TC, FC, PL, NEFA, CE & total hepatic fatty acids  ↔ hepatic TBA  ↑ cholic acid in hepatic lipid extract  ↔ serum TAG, TC, FC, HDL-C,  LDL-C, NEFA & PL  ↑ PPARα, PPARγ  Liver (mRNA):  ↑ CYP7A1 & CYP8B1, ↓ DGAT2  ↑ LXRɑ protein  ↑ fecal TBA and NEFA  Table continued overleaf | - | - | - |
| 25 | Wagnerberger, Spruss (28) | C57BL/6J mice  6-8 wo  n=4-6/group | Fructose solution (30%) | **PRO:** *L. casei* Shirota  6.8x10^7^ cfu/ g body weight  in drinking water | 8 | ↓S  (scoring: own) | ↓ALT | ↔ BW  ↔ liver wt  ↔ liver/BW ratio  ↑ calorie intake | ↓ hepatic TAG | ↔ TNFα (mRNA)  ↔ hepatic PPARγ  ↓ 4HNE protein adducts | ↔ GM of duodenal tissues  Gut permeability:  ↔ duodenal occludin  ↓ hepatic TLR 4 (mRNA) | ↔ butyrate in portal plasma |
| 26 | Kondoh, Shimada (29) | C57BL/6 mice  Male  6 wo  n=5/group | HFD (60% fat) | **PRO:** *E. faecalis* FK-23 (FK-23)  in drinking water (2% (w/w)) | 11  (9 days and 5 days interval) | ↓S  (scoring: Kleiner, Brunt (6)) | ↓ALT  ↔AST | ↔ BW  ↓ liver:BW ratio | ↔ hepatic TAG, TC, CE, NEFA, PL, diacylglycerol  ↔ serum TAG, TC, NEFA, PL  ↑ hepatic ACC, FAS  ↔ hepatic PPARɑ  ↓ FBG | ↔ TNF-α, IFN-γ &  MCP-1 | - | - |
| 27 | Li, Nie (30) | Sprague Dawley rats  Male  120–150 g  n=10/group | HFD (66.5% w/w) | a. **PRO:** *L. plantarum* NCU116  10^8^ cfu/mL  b. **PRO:** *L. plantarum* NCU116  10^9^ cfu/mL  orally | 5 | - | ↓ALT  ↓AST | ↓ adipose tissue | ↓ hepatic TAG, TC, fatty acids  ↓ FAS, ACC, SCD1  ↑ hepatic PPARɑ PPAR ɣ, PPAR δ, PGC1ɑ & CPT1ɑ  ↓ level of TBil | ↓ serum TNF-α & IL-6 (b. only)  ↑ IL10  ↑GSH-Px, CAT, T-AOC (b. only)  ↓MDA (b. only) | GM of colonic content:  ↑ Lactobacillus  ↑ Bifidobacterium  ↓Bacteroides  Gut permeability: ↓serum LPS | ↓ hepatic fatty acids |
| 28 | Reichold, Brenner (31) | C57BL/6J mice  8 wo  n=4–6/group | Western-style diet | **PRO:** *B. adolescentis*  5×10^7^ cfu/mL  in drinking water | 12 | ↓S, ↓I  (scoring: Kleiner, Brunt (6)) | ↓ALT | ↔ BW  ↔ calorie intake  ↔ liver:BW ratio | ↔ FBG | ↓ HO-1 (mRNA)  ↓ hepatic 4-HNE adducts &  iNOS (protein)  ↓ hepatic PAI-1,  CCL2, CCL19 & NFκB activity | Gut permeability:  ↑ duodenal occludin & ZO-1 (proteins)  ↔ portal endotoxin  ↓ hepatic TLR4 (protein)  ↔ MyD88 (mRNA) | - |
| 29 | Ritze, Bardos (32) | C57BL/J6 mice  Female  6 wo  n=6/group | 30% fructose solution | **PRO:** *L. rhamnosus* GG  5.2x10^7^ CFU/gram  orally | 8 | ↓S  (scoring: own) | ↓ALT | ↔ BW | ↓ hepatic TAG ↓ ChREBP, ACC1 and FAS expressions | ↓ TNF-ɑ, IL-1β & IL-8R  ↓ duodenal pIkB kinase | GM of proximal small intestinal tissue: ↔Bacteroidetes, Firmicutes  GM of distal small intestinal tissue:  ↑ total bacteria, Firmicutes &  Bacteroidetes  Gut permeability:  ↓ portal LPS  ↑ Occludin & claudin-1  ↔ ZO-1 & ZO-2 | - |
| 30 | Xin, Zeng (33) | ICR mice  Male  11±0.2g  n=6/group | HFD (80% chow, 0.5% cholesterol, 6.3% lard, 13% dried egg yolk & 0.2%  cholate)  Given chow 35D 1st (5wks 1ast) prior to HFD | a. **PRO:** *L. johnsonii* BS15 (2×10^7^ cfu/0.2 mL)  b. **PRO:** *L. johnsonii* BS15 (2×10^8^ cfu/0.2 mL)  gavage | 17 | ↓S  (scoring: modified Lee, Park (34)) | ↓ ALT | ↓ BW  ↓ perirenal, abdominal & mesenteric fats  ↓ liver wt & organ/BW | ↓ serum TAG, LDL-C and FFA  ↓ hepatic expressions of ACC1, FAS & PPARγ  ↑ FIAF  ↓ FBG, insulin & HOMA-IR | ↓ serum CRP,  ↓ hepatic TNF-α expression | Caecal content GM:  ↑ Lactobacillus spp. & Enterobacteriaceae family  ↓ Bacteroidetes/ Fermicutes ratio  Gut permeability:  ↓ serum LPS  ↓ serum 4,000 Da dextran | ↓ UCP-2 and cytochrome c in mitochondria  ↓ percentages of apoptotic cells in liver |
| 31 | Sohn, Jun (35) | C57BL/6J mice  Male  6–8 wo  n=10/group | HFD/F (40 % fat) + 10% fructose diet | **PRO:** *L. paracasei* LPC4 (KCTC 11866BP) 1 x 10^8^  gavage | 10 | ↓S, ↔I  (scoring: Yu (36)) | ↓ ALT ↔AST | ↔ BW | ↓ PPAR-γ hepatic mRNA expression | ↓ hepatic TNF-α, MCP-1 & IL-4 expressions | Gut permeability:  ↓ ^51^Cr-EDTA clearance  ↓ TLR-4 expression | ↑ F4/80^+^ CD206^+^cells  ↓ F4/80^-^CD206^-^ cells |
| 32 | Ting, Kuo (37) | Hamster  Male  8 wo  300g  n=6/group | HFD | Heat-killed:  a. **PRO:** *L. reuteri* GMNL-263 (625 mg/kg-hamster/day)  b. **PRO:** *L. reuteri* GMNL-263 (3125 mg/kg-hamster/day)  gavage | 8 | ↓S, ↓F  (scoring: own) | - | ↓ BW & epididymal AT wt  (b. only)  ↔ food intake | ↓ hepatic TAG, faecal TAG and faecal TC (b. only)  ↓FAS  ↔ HMG-CoA reductase, PPARγ  ↑ CYP7A1 and LDLR | ↓ hepatic TGF-β  ↓ hepatic MDA | - | Improved heart function; fractional shortening & ejection fraction  (echo) |
| 33 | Ye, Li (38) | Wistar rats  Male  200±10 g  n=8/group | HFD (60% fat) | a. **PRO:** *L. paracasei* Jlus66 1×10^10^ cfu b. **PRO:** *L. paracasei* Jlus66 2×10^10^ cfu c. **PRO:** *L. paracasei* Jlus66 4×10^10^ cfu  orally | 20 | ↓S, ↓I  (scoring: Kleiner, Brunt (6) with modification) | ↓ ALT  (c. only)  ↔AST | ↓ BW (c. only)  ↓ liver wt  ↓ liver:BW ratio | ↓ serum TAG (c. only) ↓ serum LDL-C (b. and c. only)  ↔ serum TC, HDL-C | -  Table continued overleaf | - | - |
| 34 | Chen, Lin (39) | Sprague Dawley rats  Male  6 wo  n=12/group | HFD (60% fat) | a. **PRO:** *L. mali* APS1 10^7^ CFU/mouse  b. **PRO:** *L. mali* APS1 10^8^ CFU/mouse  c. **PRO:** *L. mali* APS1 10^9^ CFU/mouse  gavage | 12 | ↓S  (scoring: own) | ↔ ALT  ↔ AST | ↓ BW gain  (b. only) | ↑ Sirt-1 (b. only)  ↑ PGC-1α , ↔SREBP-1  ↓ FBG (b and c. only)  ↓ HOMA-IR (b. and c. only)  ↑ GLP-1 (b. and c. only) | ↓ MDA, ↑SOD, ↔ CAT  ↑ Nrf-2 (protein)  ↔ HO-1 | GM of caecal content:  relative abundance of ↓Firmicutes & Verrucomicrobia | Ceacal content SCFA:  ↔ butyrate |
| 35 | Liang, Lin (40) | Sprague Dawley rats  Male  6 wo  n=5/group | HFD (83% basal feed + 10% lard oil + 5% sucrose + 1.5% cholesterol + 0.5% cholate) | **PRO:** Mixture of 6 *Lactobacillus* and 3 *Bifidobacterium*  6 billion cfu/g | 16 | ↓S  (scoring: own) | ↓ ALT  ↔ AST  ↔ ALP | ↓ BW gain  ↔ food intake | ↓ serum TAG, FFA  ↔ TC, HDL-C, LDL-C  ↔ FBG, insulin and HOMA-IR | ↓ serum IL-1β & IL-18  ↔ serum TNF-ɑ | - | ↑ faecal butyrate  ↔ faecal isobutyrate  ↓ hepatic & AT Gpr109a expression |
| 36 | Park, Lee (41) | C57BL/6 mice  Male  7 wo  19 ± 2 g  n=4-9/group | HFD (45% fat) | **PRO:** *L. acidophilus* NS1 (LNS1) ~1.0 × 10^8^ cfu/mL  orally | 12 | ↓S  (scoring: own) | - | ↓ BW gain  ↓ liver wt  ↓ inguinal & epididymal AT  ↔ feed intake | ↓ hepatic fatty acid  ↑ hepatic fatty acid oxidation  ↑ serum adiponectin & ↓ leptin    ↓ hepatic and AT SREBP-1c, FAS and ACC ↑ SREBP-2 (mRNA) ↑ AMPK activity  ↓ FBG, insulin levels, GTT | ↓serum TNFα  ↑ hepatic and AT PPARɑ, ACOX  & CPT1 | - | - |
| 37 | Werawatganon, Somanawat (42) | Sprague Dawley rats  Male  220–250 g  n=8/group | 100% fat | **PRO:** *L. plantarum* 1.8 × 10^9^ CFUs/mL; twice a day | 6 | ↓S, ↓I  (scoring: Bacon, Farahvash (43)) | - | - | ↑ PPAR-γ positive stained cells | ↓ serum TNF-ɑ  ↓ hepatic MDA | - | - |
| 38 | Jang, Park (44) | C57BL/6J mice  Male  13 & 5 wo | HFD (60%)  HFD (45%) | a. **PRO:** live *L. rhamnosus* GG  1 × 10^9^ cfu/mouse /day  b. **PRO:** formalin-killed *L. rhamnosus* GG  1 × 10^9^ cfu/mouse /day  gavage | 19 | - | - | - | - | - | - | - |
| 39 | Wang, Li (45) | Wistar rats  Male, SPF  (200 ± 10 g)  n=8/group | HFD (360 g/kg fat) | a. **PRO:** *L. paracasei* Jlus66 1× 10^10^ cfu  b. **PRO:** *L. paracasei* Jlus66 2× 10^10^ cfu  c. **PRO:** *L. paracasei* Jlus66 4× 10^10^ cfu  orally | 20 | - | - | - | - | ↑ SOD & GSH-Px  ↑ IL-10 (except a.)  ↓ TNF-α, IL-6  ↓ MDA (except a.) | GM of caecal content:  ↑diversity (c. only)  ↑ Firmicutes &  ↓ Bacteroidetes (c. only)  ↑ Firmicutes:Bacteroidetes ratio (c. only)    Gut permeability:  ↓ serum LPS (except a.) | - |
| 40 | Wang, Xu (46) | C57BL/6N mice  Male  ~18.5 g  n=10/group | HFD | **PRO:** *L. plantarum* X (1× 10^8^ CFU/ml) + *B. bifidum* V (2× 10^8^ CFU/ml)  gavage | 6 | - | ↓ALT  ↓AST | ↓ BW gain  ↔ food intake  ↓ liver wt | ↓ hepatic TAG, TC  ↓ serum TAG, TC, LDL-C  ↑ serum HDL-C & ↑ faecal TC  ↔ FBG, serum insulin, OGTT  ↓ HOMA-IR | ↓ hepatic TNF-ɑ,  IL-6 & IL-1β | GM of intestinal content:  ↑ Bacteroidetes/Firmicutes ratio  ↑ Bacteroides, Lactobacillus, & Parabacteroides  Gut permeability: ↓serum LPS | Caecal SCFA:  ↑ butyric and acetic acids |
| 41 | Zhao, Wang (47) | C57BL/6J mice  Male  8 wo  n=10/group | HFD/F  (65% fat & 30% fructose solution) | **PRO:** *L. plantarum* NA136 1.0×10^9^ CFU/day/mice  orally | 16 | ↓S, ↓I  (scoring: own) | ↓ALT  ↓AST | ↔ BW | ↓ serum TAG, TC, LDL-C, HDL-C  ↓ serum and hepatic FFA  ↑ hepatic AMPK & ACC phosphorylation  ↓ hepatic SREBP-1 & FAS | ↑ hepatic CAT & SOD  ↓ hepatic MDA  ↑ hepatic HO-1 & Nrf2  ↓ hepatic Keap-1 | - | - |
| 42 | Kim, Lee (48) | C57BL/6N mice  Male  5 wo  n=5/group | HFD (45% fat) | **PRO:** *A. muciniphila* 10^8^-10^9^ CFU/mL  gavage | 10 | ↓S  (scoring:  Keenan, Baker (49) | ↓ALT  ↓AST | ↔ BW gain | ↓ serum TAG  ↓ SREBP, ↔ ChREBP | ↓IL-6 | GM of intestinal content:  ↓ Bacteroidetes  ↑ Firmicutes  ↑ *Pseudoflavonifractor*  Table continued overleaf |  |
| 43 | Lee, Yoon (50) | C57BL/6J mice  Male  6 wo  n=10/group | Western diet  (42% fat, 42.7% of carbohydrate, and 15% protein) | a. **PRO:** *L. bulgaricus* 10^9^ CFU/g  b. **PRO:** *L. casei* 10^9^ CFU/g  c. **PRO:** *L. helveticus* 10^9^ CFU/g  d. **PRO:** *P. pentosaceus* KID7 10^9^ CFU/g  e. **PRO*:*** *L. casei*+*L. helveticus* 10^9^ CFU/g  f. **PRO:** *L. casei*+*L. helveticus*  *+P. pentosaceus* KID7 10^9^ CFU/g  g. **PRO:** *L. casei*+*L. helveticus+L.*  *bulgaricus* 10^9^ CFU/g  gavage | 8 | ↓S  (except b.)  (scoring: Kleiner, Brunt (6)) | ↓ALT  (a. only)  ↓AST  (a. only) | ↓ liver/BW ratio (a., c., d., e. and f. only) | ↓serum TC  ↓ CD68 macrophage marker in liver  (a., b., c., d. only) | ↓TNF-ɑ  ↓ IL-1ꞵ (except e. & f.)  ↓ IL-6 (except e.) | GM of caecum:  ↓ Firmicutes: Bacteroidetes ratio (a., b., c., d. only)  ↑ *A. muciniphila*  (a., b. and c.only)  ↓ stool endotoxin  (a., b., c., d. only) | - |
| 44 | Yao, Jia (51) | C57BL/6 mice  Male  4 wo  n=10/group | HFD | **PRO:** *L. paracasei* N1115, 2.2 × 10^9^  CFU/ml in normal saline, 0.5 ml/day  Administration not mentioned | 16 | ↓S  (scoring: Takahashi and Fukusato (52)) | - | - | ↓ serum TAG, TC  ↓ FBG, fasting insulin & HOMA-IR  ↔ IPGTT  ↔ hepatic insulin receptor mRNA  & hepatic insulin receptor substrate-1 mRNA | ↓ serum and hepatic TNF-α, IL-1ꞵ  ↓ hepatic NF-Kꞵ  ↓ serum monoamine oxidase (MAO) | Gut permeability:  ↓ serum LPS & hepatic TLR4  Improved intestinal barrier function (p-p38, occludin-1 and claudin-1) |  |

**Legend:**

Table legend continued overleaf

Table legend continued overleaf

| **†** – NAFLD induction conducted prior to intervention | – n in accordance to the material / methodology | **Note:** gavage covers administration techniques with the similar principle such as intragastric tube or *sonde* |
| --- | --- | --- |
| **Interventions:** |  |  |
| *A. – Akkermansia*  *B.* – *Bifidobacterium*  Bacillus mixture – *B. sonorensis* JJY12-3, *B. paralicheniformis* JJY12-8, *B. sonorensis* JJY13-1, *B. sonorensis* JJY 13–3, and *B. sonorensis* JJY 13–8  *C. – Clostridium*  *E.* – *Enterococcus*  *E. coli* – *Escherichia coli*  *L.* – *Lactobacillus* | *P. – Pediococcus*  Probiatop® – 10^9^ CFU of each probiotic strain (*L. paracasei* Lpc-37 SD 5275, *L. rhamnosus* HN001 SD 5675, *L. acidophilus* NCFM SD5221, *B. lactis* HN019 SD 5674)  *S.* – *Streptococcus*  Symbiter - multiprobiotic contain: 14 probiotic bacteria of genera Bifidobacterium, Lactobacillus, Lactococcus, Propionibacterium  VSL#3 – mixure of 4 strains of *Lactobacillus*, 3 strains of *Bifidobacteria* and *S. thermophilus* | VSL#3+ – Bifidobacteria, Lactobacilli and *S. thermophilus*  VSL#3++ – 3.1% (wt:wt) of *L. plantarum*,7.3% of *L. acidophilus*,16% of *L. casei*, and 8.4% *of L. delbrueckii* subsp*. bulgaricus*; *B. infantis*, *B.* *breve* and *B. longum* (17.7%) and 47.5% of *S. salivarius* subsp. *thermophilus*  VSL#3+++ – from VSL Pharmateuticals, Ft. Lauderdale, FL (450 billion colonies/packet)  VSL#3++++ – mixture of viable, lyophilized Bifidobacteria, Lactobacilli and *S. thermophilus*): 1.5 x 10^9^ colonies/mouse/day or low-dose (1.5 x 10^8^ colonies/mouse/day), single-strain probiotic (*B. infantis*) |
|  |  |  |
| **General:** | **Induction:** | **Histology:** |
| BW – body weight | AIN - American Institute Nutrition | B – ballooning |
| Da – Daltons | CDAA – choline-deficient/L-amino acid-defined diet | F – fibrosis |
| MSG – monosodium glutamate | HFCD – high-fat-choline-deficient | I – inflammation |
| wt – weight | HFD – high-fat diet | IS – immunostaining |
| s.c. – sub-cutaneous | ND – normal diet | S – steatosis |
| wo – weeks old |  |  |
|  |  |  |
| **Biochemical / molecular:** |  |  |
| ACC – acetyl-CoA carboxylase | GTT – glucose tolerance test | NKT – natural killer T cell |
| Acc 1 – acetyl-CoA carboxylase 1 | GSH-Px – glutathione peroxidase | Nrf2 – nuclear factor erythroid 2-related factor 2 |
| Acox1 – genes related to fatty acid oxidation | gWAT – gonadal white adipose tissue | OGTT – oral glucose tolerance test |
| ALP – alkaline phosphatase | HDL-C – High density lipoprotein cholesterol | PGC-1ɑ – peroxisome proliferator-activated receptor gamma (PPAR-ɣ) coactivator 1 alpha |
| ALT – alanine aminotransferase | HOMA-IR – homeostasis model assessment for insulin resistance | PL – phospholipid |
| AMPK – AMP-activated protein kinase | HMG-CoA – 3-hydroxy-3-methylglutaryl coenzyme A | PPARɑ – peroxisome proliferator-activated receptor alpha |
| AST – aspartate aminotransferase | HMGCR – 3-hydroxy-3-methyl-glutaryl-CoA reductase | PPARδ – peroxisome proliferator-activated receptor delta |
| ASC– apoptosis-associated speck-like protein | HMGCS – HMG-CoA synthase | PPARɣ – peroxisome proliferator-activated receptor gamma |
| AT – adipose tissue | HRSA – hydroxyl radical suppress activity | proMMP-9 – pro-matrix metalloproteinase 9 |
| BAT – brown adipose tissue | 4-HNE – 4-hydroxynonenal | Reg3ɣ – regenerating islet-derived protein 3 gamma |
| BDNF – brain-derived neurotrophic factor | IFN-γ – interferon-gamma | SCD-1 – stearoyl-coenzyme A desaturase 1 |
| CASPASE-1 – proinflammatory enzyme caspase-1 | IKK-ꞵ – inhibitor kappa kinase beta | SCFA – short-chain fatty acid |
| CAT – catalase | IL-4 – interleukin 4 | SHP – Small heterodimer partner |
| CCL17 – C-C chemokine ligand 17 | IL-6 – interleukin 6 | SOCS3 – suppressor of cytokine signaling3 |
| CCL20 – C-C chemokine ligand 20 | IL-1ꞵ – interleukin 1 beta | SOD – superoxide dismutase |
| CE – cholesterol ester | IL-10 – interleukin 10 | SR-B1 – Scavenger receptor class B type 1 |
| ChREBP – carbohydrate response-element binding protein | IL-12B – interleukin 12B | SREBP – Sterol-regulatory element binding protein |
| COL1A1 – Collagen Type I Alpha 1 Chain | IL-17 – interleukin 17 | TAC – total antioxidant capacity |
| COX-2 – cyclooxygenase-2 | IL-22 – interleukin 22 | TAG – triacylglycerol |
| CPT-1 – carnitine palmitoyltransferase I | iNOS – inducible nitric oxygen synthase | TBA – total bile acid |
| ^51^Cr-EDTA – ^51^Cr-Ethylenediaminetetraacetic acid | IPGTT – intraperitoneal glucose tolerance tests | TC – total cholesterol |
| CRP – C-reactive protein | IR – insulin resistance | TGF-ꞵ – transforming growth factor beta |
| CXCL10 – C-X-C motif chemokine ligand 10 | ISI – insulin sensitive index | TGR5 – Takeda G-protein-coupled receptor 5 |
| CYP7A1 – cholesterol 7-α-hydroxylase | Keap-1 – Kelch ECH associating protein 1 | TIMP1 – tissue inhibitor of matrix metalloproteinases-1 |
| CYP8B1 – sterol 12ɑ-hydroxylase | LDLR – low density lipoprotein receptor | TOC – total oxidant status |
| DGAT2 – diacylglycerol acyltransferase 2 | LDL-C – Low density lipoprotein cholesterol | TLCA – taurolithocholic acid |
| EAT – epididymal adipose tissue | LPS – lipopolysaccharide | UCP-2 – uncoupling protein 2 |
| EC – esterified cholesterol | LXRɑ – liver-X-receptor | Tbil – total bilirubin |
| FAS – fatty acid synthase | MAT – mesenteric adipose tissue | TLR2 – toll-like receptor 2 |
| FBG – fasting blood glucose | MCP-1 – monocyte chemotactic and activating factor 1 | TLR4 – toll-like receptor 4 |
| FC – free cholesterol | MDA – malondialdehyde | TLR9 – toll-like receptor 9 |
| FGF21 – fibroblast growth factor 21 | MMPs – matrix metalloproteinases | TNFɑ – tumour necrosis factor ɑ |
| FGFR4 – Fibroblast Growth Factor Receptor 4 | MMP-2 – matrix metalloproteinase 2 | VLDL – very low-density lipoprotein |
| FIAF – fasting-induced adipose factor | MUFA – Mono-unsaturated fatty acid | WAT – white adipose tissue |
| FITC – fluorescein isothiocyanate | Myd88 - myeloid differentiation factor 88 | ZO-1 – zonula occludens 1 |
| Foxp3 – forkhead box P3 | NEFA – non-esterified fatty acid | ZO-2 – zonula occludens 2 |
| FXR – farnesoid X receptor | NFкB – necrosis factor kappa B |  |
| GLP-1 – glucagon-like peptide-1 | NF-κβ – transcription factor nuclear factor-kappa beta |  |

**References**

1. Ma X, Hua J, Li Z. Probiotics improve high fat diet-induced hepatic steatosis and insulin resistance by increasing hepatic NKT cells. J Hepatol. 2008;49(5):821-30.

2. Li Z, Yang S, Lin H, Huang J, Watkins PA, Moser AB, et al. Probiotics and antibodies to TNF inhibit inflammatory activity and improve non-alcoholic fatty liver disease. Hepatology. 2003;37(2):343-50.

3. Velayudham A, Dolganiuc A, Ellis M, Petrasek J, Kodys K, Mandrekar P, et al. VSL#3 probiotic treatment attenuates fibrosis without changes in steatohepatitis in a diet-induced non-alcoholic steatohepatitis model in mice. Hepatology. 2009;49(3):989-97.

4. Bhathena J, Martoni C, Kulamarva A, Tomaro-Duchesneau C, Malhotra M, Paul A, et al. Oral probiotic microcapsule formulation ameliorates non-alcoholic fatty liver disease in Bio F1B Golden Syrian hamsters. PLoS One. 2013;8(3):e58394.

5. Savcheniuk O, Kobyliak N, Kondro M, Virchenko O, Falalyeyeva T, Beregova T. Short-term periodic consumption of multiprobiotic from childhood improves insulin sensitivity, prevents development of non-alcoholic fatty liver disease and adiposity in adult rats with glutamate-induced obesity. BMC Complement Altern Med. 2014;14:247.

6. Kleiner DE, Brunt EM, Van Natta M, Behling C, Contos MJ, Cummings OW, et al. Design and validation of a histological scoring system for nonalcoholic fatty liver disease. Hepatology. 2005;41(6):1313-21.

7. Liang S, Webb T, Li Z. Probiotic antigens stimulate hepatic natural killer T cells. Immunology. 2014;141(2):203-10.

8. Ivanovic N, Minic R, Dimitrijevic L, Radojevic Skodric S, Zivkovic I, Djordjevic B. *Lactobacillus rhamnosus* LA68 and *Lactobacillus plantarum* WCFS1 differently influence metabolic and immunological parameters in high fat diet-induced hypercholesterolemia and hepatic steatosis. Food Funct. 2015;6(2):558-65.

9. Briskey D, Heritage M, Jaskowski LA, Peake J, Gobe G, Subramaniam VN, et al. Probiotics modify tight-junction proteins in an animal model of non-alcoholic fatty liver disease. Therap Adv Gastroenterol. 2016;9(4):463-72.

10. Kobyliak N, Falalyeyeva T, Virchenko O, Mykhalchyshyn G, Bodnar P, Spivak M, et al. Comparative experimental investigation on the efficacy of mono- and multiprobiotic strains in non-alcoholic fatty liver disease prevention. BMC Gastroenterol. 2016;16(1):34.

11. Kim B, Park KY, Ji Y, Park S, Holzapfel W, Hyun CK. Protective effects of *Lactobacillus rhamnosus* GG against dyslipidemia in high-fat diet-induced obese mice. Biochem Biophys Res Commun. 2016;473(2):530-6.

12. Zhou D, Pan Q, Liu XL, Yang RX, Chen YW, Liu C, et al. *Clostridium butyricum* B1 alleviates high-fat diet-induced steatohepatitis in mice via enterohepatic immunoregulation. Clin Gastroenterol Hepatol. 2017;32(9):1640-8.

13. Jena PK, Sheng L, Nagar N, Wu C, Barile D, Mills DA, et al. Synbiotics *Bifidobacterium infantis* and milk oligosaccharides are effective in reversing cancer-prone non-alcoholic steatohepatitis using western diet-fed FXR knockout mouse models. J Nutr Biochem. 2018;57:246-54.

14. Kobyliak N, Abenavoli L, Falalyeyeva T, Beregov T. Efficacy of probiotics and smectite in rats with non-alcoholic fatty liver disease. Ann Hepatol. 2018;17(1):153-61.

15. dos Santos AC, Ribeiro DA, Ferreira JAD, Aguiar O, Waitzberg DL, Alves CC. Inflammatory gene expression analysis after prebiotic, probiotic and synbiotic supplementation in experimental nonalcoholic fatty liver disease. Nutr Food Sci. 2019;49(1):75-84.

16. Zhao C, Liu L, Liu Q, Li F, Zhang L, Zhu F, et al. Fibroblast growth factor 21 is required for the therapeutic effects of *Lactobacillus rhamnosus* GG against fructose-induced fatty liver in mice. Mol Metab. 2019;29:145-57.

17. Wang Y, Kirpich I, Liu Y, Ma Z, Barve S, McClain CJ, et al. *Lactobacillus rhamnosus* GG treatment potentiates intestinal hypoxia-inducible factor, promotes intestinal integrity and ameliorates alcohol-induced liver injury. Am J Pathol. 2011;179(6):2866-75.

18. Mohammed SK, Magdy YM, El-Waseef DA, Nabih ES, Hamouda MA, El-Kharashi OA. Modulation of hippocampal TLR4/BDNF signal pathway using probiotics is a step closer towards treating cognitive impairment in NASH model. Physiol Behav. 2020;214:112762.

19. Park EJ, Lee YS, Kim SM, Park GS, Lee YH, Jeong DY, et al. Beneficial effects of *Lactobacillus plantarum* strains on non-alcoholic fatty liver disease in high fat/high fructose diet-fed rats. Nutrients. 2020;12(2).

20. Rashid H, Khan JA, Muhammad F, Abbas RZ. Probiotics supplementation reduces high fat high sugar diet-associated oxidative stress at intestinal epithelial cells, nephrons and hepatocytes in rat model. Int J Agric Biol. 2020;23(2):454-62.

21. Esposito E, Iacono A, Bianco G, Autore G, Cuzzocrea S, Vajro P, et al. Probiotics reduce the inflammatory response induced by a high-fat diet in the liver of young rats. J Nutr. 2009;139(5):905-11.

22. Karahan N, Isler M, Koyu A, Karahan AG, Kilic GB, Ciris IM, et al. Effects of probiotics on methionine choline-deficient diet-induced steatohepatitis in rats. Turk J Gastroenterol. 2012;23(2):110-21.

23. Sundaram SS, Whitington PF, Green RM. Steatohepatitis develops rapidly in transgenic mice overexpressing Abcb11 and fed a methionine-choline-deficient diet. Am J Physiol Gastrointest Liver Physiol 2005;288(6):G1321-7.

24. Xu RY, Wan YP, Fang QY, Lu W, Cai W. Supplementation with probiotics modifies gut flora and attenuates liver fat accumulation in rat non-alcoholic fatty liver disease model. J Clin Biochem Nutr. 2012;50(1):72-7.

25. Endo H, Niioka M, Kobayashi N, Tanaka M, Watanabe T. Butyrate-producing probiotics reduce non-alcoholic fatty liver disease progression in rats: new insight into the probiotics for the gut-liver axis. PLoS One. 2013;8(5):e63388.

26. Okubo H, Sakoda H, Kushiyama A, Fujishiro M, Nakatsu Y, Fukushima T, et al. L*actobacillus casei* strain Shirota protects against non-alcoholic steatohepatitis development in a rodent model. Am J Physiol Gastrointest Liver Physiol. 2013;305(12):G911-G8.

27. Seo M, Inoue I, Tanaka M, Matsuda N, Nakano T, Awata T, et al. *Clostridium butyricum* MIYAIRI 588 improves high-fat diet-induced non-alcoholic fatty liver disease in rats. Dig Dis Sci. 2013;58(12):3534-44.

28. Wagnerberger S, Spruss A, Kanuri G, Stahl C, Schröder M, Vetter W, et al. *Lactobacillus casei* Shirota protects from fructose-induced liver steatosis: a mouse model. J Nutr Biochem. 2013;24(3):531-8.

29. Kondoh M, Shimada T, Fukada K, Morita M, Katada K, Higashimura Y, et al. Beneficial effects of heat-treated *Enterococcus faecalis* FK-23 on high-fat diet-induced hepatic steatosis in mice. Br J Nutr. 2014;112(6):868-75.

30. Li C, Nie SP, Zhu KX, Ding Q, Li C, Xiong T, et al. *Lactobacillus plantarum* NCU116 improves liver function, oxidative stress and lipid metabolism in rats with high fat diet induced non-alcoholic fatty liver disease. Food Funct. 2014;5(12):3216-23.

31. Reichold A, Brenner SA, Spruss A, Förster-Fromme K, Bergheim I, Bischoff SC. *Bifidobacterium adolescentis* protects from the development of non-alcoholic steatohepatitis in a mouse model. J Nutr Biochem 2014;25(2):118-25.

32. Ritze Y, Bardos G, Claus A, Ehrmann V, Bergheim I, Schwiertz A, et al. *Lactobacillus rhamnosus* GG protects against non-alcoholic fatty liver disease in mice. PLoS One. 2014;9(1):e80169.

33. Xin J, Zeng D, Wang H, Ni X, Yi D, Pan K, et al. Preventing non-alcoholic fatty liver disease through *Lactobacillus johnsonii* BS15 by attenuating inflammation and mitochondrial injury and improving gut environment in obese mice. Appl Microbiol Biotechnol. 2014;98(15):6817-29.

34. Lee HY, Park JH, Seok SH, Baek MW, Kim DJ, Lee KE, et al. Human originated bacteria, *Lactobacillus rhamnosus* PL60, produce conjugated linoleic acid and show anti-obesity effects in diet-induced obese mice. Biochim Biophys Acta. 2006;1761(7):736-44.

35. Sohn W, Jun DW, Lee KN, Lee HL, Lee OY, Choi HS, et al. *Lactobacillus paracasei* induces M2-dominant kupffer cell polarization in a mouse model of non-alcoholic steatohepatitis. Dig Dis Sci. 2015;60(11):3340-50.

36. Yu E. [Histologic grading and staging of chronic hepatitis: on the basis of standardized guideline proposed by the Korean Study Group for the Pathology of Digestive Diseases]. Korean J Hepatol. 2003;9(1):42-6.

37. Ting WJ, Kuo WW, Hsieh DJ, Yeh YL, Day CH, Chen YH, et al. Heat killed *Lactobacillus reuteri* GMNL-263 reduces fibrosis effects on the liver and heart in high fat diet-hamsters via TGF-beta suppression. Int J Mol Sci. 2015;16(10):25881-96.

38. Ye H, Li Q, Zhang Z, Sun M, Zhao C, Zhang T. Effect of a novel potential probiotic *Lactobacillus paracasei* Jlus66 isolated from fermented milk on non-alcoholic fatty liver in rats. Food Funct. 2017;8(12):4539-46.

39. Chen YT, Lin YC, Lin JS, Yang NS, Chen MJ. Sugary kefir strain *Lactobacillus mali* APS1 ameliorated hepatic steatosis by regulation of SIRT-1/Nrf-2 and gut microbiota in rats. Mol Nutr Food Res. 2018;62(8):e1700903.

40. Liang Y, Lin C, Zhang Y, Deng Y, Liu C, Yang Q. Probiotic mixture of *Lactobacillus* and *Bifidobacterium* alleviates systemic adiposity and inflammation in non-alcoholic fatty liver disease rats through Gpr109a and the commensal metabolite butyrate. Inflammopharmacology. 2018:1-5.

41. Park SS, Lee YJ, Song S, Kim B, Kang H, Oh S, et al. *Lactobacillus acidophilus* NS1 attenuates diet-induced obesity and fatty liver. J Endocrinol. 2018;237(2):87-100.

42. Werawatganon D, Somanawat K, Tumwasorn S, Klaikeaw N, Siriviriyakul P. *Lactobacillus plantarum* attenuates oxidative stress and liver injury in rats with non-alcoholic steatohepatitis. Pharmacogn Mag. 2018;14(58):471-6.

43. Bacon BR, Farahvash MJ, Janney CG, Neuschwander-Tetri BA. Nonalcoholic steatohepatitis: an expanded clinical entity. Gastroenterology. 1994;107(4):1103-9.

44. Jang HR, Park HJ, Kang D, Chung H, Nam MH, Lee Y, et al. A protective mechanism of probiotic *Lactobacillus* against hepatic steatosis via reducing host intestinal fatty acid absorption. Exp Mol Med. 2019;51.

45. Wang W, Li Q, Chai WH, Sun CY, Zhang TH, Zhao CH, et al. *Lactobacillus paracasei* Jlus66 extenuate oxidative stress and inflammation via regulation of intestinal flora in rats with non-alcoholic fatty liver disease. Food Sci Nutr. 2019;7(8):2636-46.

46. Wang W, Xu AL, Li ZC, Li Y, Xu SF, Sang HC, et al. Combination of probiotics and *Salvia miltiorrhiza* polysaccharide alleviates hepatic steatosis via gut microbiota modulation and insulin resistance improvement in high fat-induced NAFLD mice. Diabetes Metab J. 2019.

47. Zhao Z, Wang C, Zhang L, Zhao Y, Duan C, Zhang X, et al. *Lactobacillus plantarum* NA136 improves the non-alcoholic fatty liver disease by modulating the AMPK/Nrf2 pathway. Appl Microbiol Biotechnol. 2019;103(14):5843-50.

48. Kim S, Lee Y, Kim Y, Seo Y, Lee H, Ha J, et al. *Akkermansia muciniphila* prevents fatty liver, decreases serum triglycerides, and maintains gut homeostasis. Appl Environ Microbiol. 2020;17.

49. Keenan CM, Baker J, Bradley A, Goodman DG, Harada T, Herbert R, et al. International Harmonization of Nomenclature and Diagnostic Criteria (INHAND): Progress to Date and Future Plans. Toxicologic pathology. 2015;43(5):730-2.

50. Lee NY, Yoon SJ, Han DH, Gupta H, Youn GS, Shin MJ, et al. *Lactobacillus* and *Pediococcus* ameliorate progression of non-alcoholic fatty liver disease through modulation of the gut microbiome. Gut Microbes. 2020:1-18.

51. Yao F, Jia R, Huang H, Yu Y, Mei L, Bai L, et al. Effect of *Lactobacillus paracasei* N1115 and fructooligosaccharides in non-alcoholic fatty liver disease. Arch Med Sci. 2019;15(5):1336-44.

52. Takahashi Y, Fukusato T. Histopathology of nonalcoholic fatty liver disease/nonalcoholic steatohepatitis. World J Gastroenterol. 2014;20(42):15539-48.
